# Supplementary material for: Heterologous arenavirus vector prime-boost overrules self-tolerance for efficient tumor-specific CD8 T cell attack
Source: Cell Rep Med. 2021 Mar 3;2(3):100209. doi: 10.1016/j.xcrm.2021.100209 (PMC7974551; doi:10.1016/j.xcrm.2021.100209)
Supplement: Document S1. Figures S1–S7 [file mmc1.pdf]

Cell Reports Medicine, Volume *2*

Supplemental information

Heterologous arenavirus vector prime-boost overrules self-tolerance for efficient tumor-specific CD8 T cell attack

Weldy V. Bonilla, Nicole Kirchhammer, Anna-Friederike Marx, Sandra M. Kallert, Magdalena A. Krzyzaniak, Min Lu, Stéphanie Darbre, Sarah Schmidt, Josipa Raguz, Ursula Berka, Ilena Vincenti, Mindaugas Pauzuolis, Romy Kerber, Sabine Hoepner, Stephan Günther, Carsten Magnus, Doron Merkler, Klaus K. Orlinger, Alfred Zippelius, and Daniel D. Pinschewer

Supplementary Figure 1

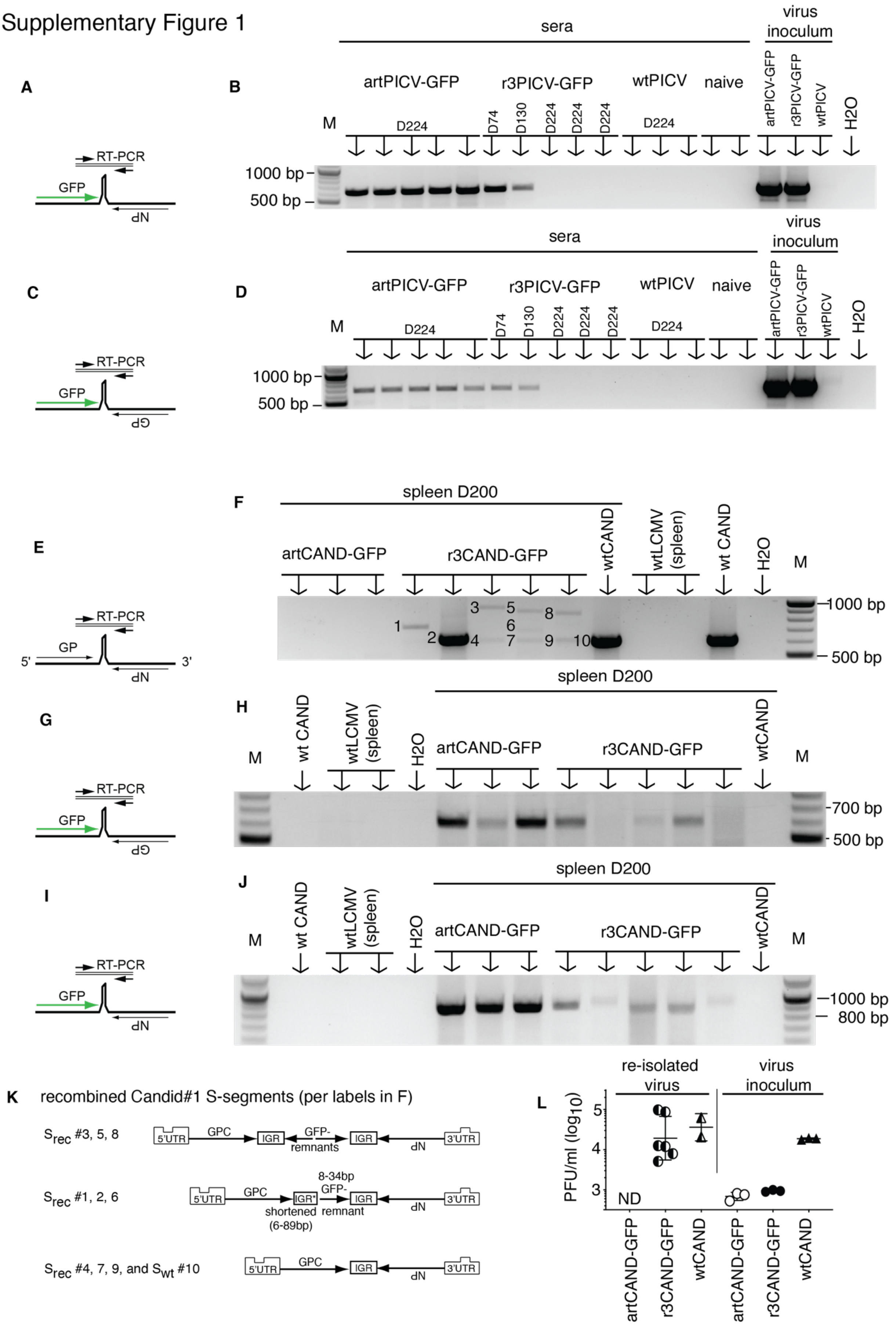

Figure S1: Genetic and phenotypic stability of artARENA and r3ARENA vectors. Related to Figure 1.

A-D: RT-PCR strategy (A) to amplify non-recombined S_{NP} (B) and RT-PCR strategy (C) to amplify S_{GP} segment (D) RNA species of both artPICV-GFP and r3PICV-GFP from the samples of the experiment shown in Fig. 1H-N. Gel electrophoresis image of RT-PCR products (B,D) obtained from serum samples collected on the indicated time points. Each lane represents an individual mouse. Sera from uninfected mice ("naive"), from three wtPICV infected mice and water were included as negative controls. Virus inocula served as reference.

E-L: We infected AGRAG mice with artCAND-GFP, r3CAND-GFP or CANDwt and collected spleen tissue 200 days later.

E,F: RT-PCR strategy (E) applied to amplify recombined wildtype-like CAND S segment RNA species re-uniting NP and GP sequences (F). Gel electrophoresis image of RT-PCR products (F) obtained from spleen samples collected on the indicated time points after infection of AGRAG mice. Water and spleen tissue of AGRAG mice infected with LCMVwt served as negative control. Spleen tissue from a wtCAND-inoculated AGRAG mouse and wtCAND inoculum virus served as positive controls. Each lane with a spleen sample represents an individual mouse.

G-J: RT-PCR strategy (G) to amplify non-recombined S_{GP} (H) and RT-PCR strategy (I) to amplify S_{NP} segment (J) RNA species of both artCAND-GFP and r3CAND-GFP. Gel electrophoresis image of RT-PCR products (H,J) obtained from spleen samples collected on d200. Each lane represents an individual mouse. Spleen tissue from AGRAG mice infected with wtCAND or wtLCMV as well as wtCAND virus from cell culture and water were included as negative controls in the assay.

K: Sequence analysis of the bands numbered in (F) suggested viral recombination products as schematically depicted in (K). IGR: S segment intergenic region; IGR*: truncated S segment intergenic region; UTR: untranslated region

L: 293T cells were infected at MOI=0.001 with viruses re-isolated from individual AGRAG mice on d200 of the experiment in (E-K) or with the inoculum used to infect the animals. Titers after 72h are shown. Symbols represent individual viral cultures from one mouse each (re-isolated virus) or from replicate cultures (virus inoculum). Mean±SD is indicated. We failed to re-isolate infectious virus from artCAND-infected mice (ND).

Supplementary Figure 2

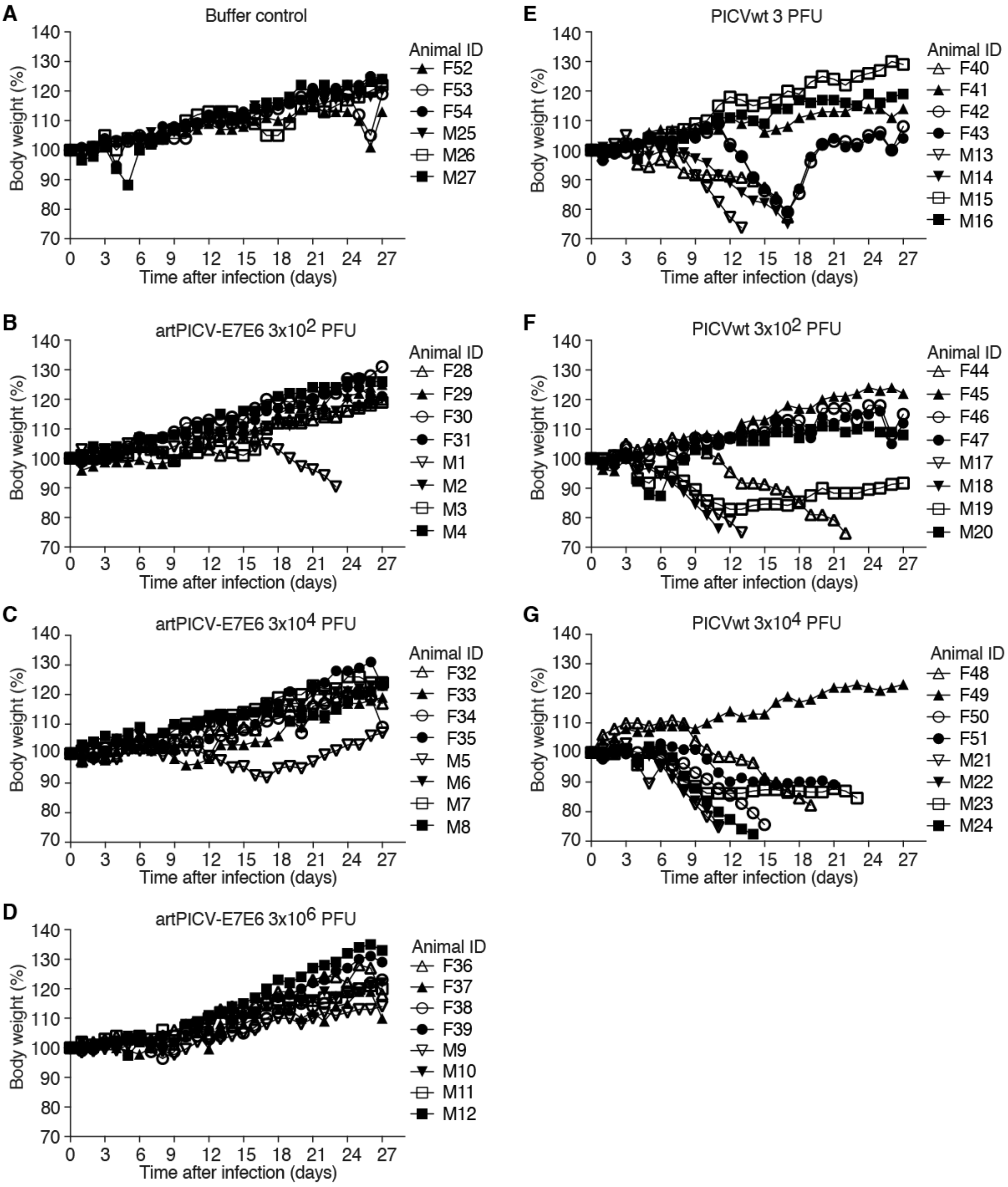

Figure S2: artARENA vectors are attenuated in guinea pigs. Related to Figure 2.

We infected groups of 8 adult Hartley guinea pigs, four of each sex (F: female; M: male), with either artPICV-E7E6 or PICVwt at the indicated doses intraperitoneally and monitored body weight over time. A group of six control animals (three of each sex) was administered diluent. Data are from the same experiment as in Fig. 2A-H.

Supplementary Figure 3

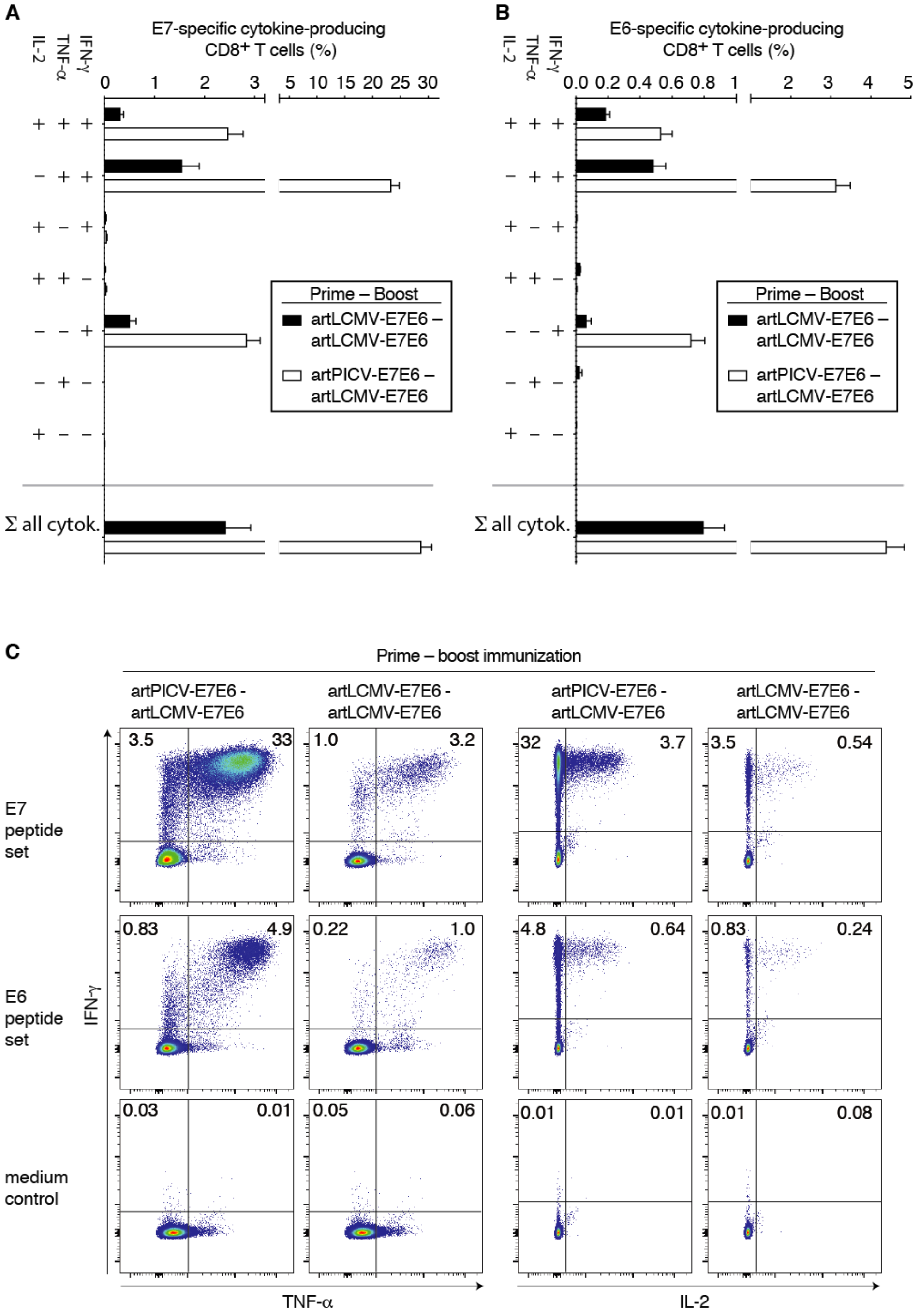

Figure S3: Functionality of CD8 T cell response to heterologous artARENA prime – boost vaccination. Related to Figure 3.

A-E: On d0 and d13 C57BL/6 mice were given i.v. vaccination with artPICV-E7E6 and artLCMV-E7E6, either as homologous or heterologous prime – boost, as indicated. On d51 the animals were sacrificed to determine E7- (A) and E6-specific (B) cytokine-secreting cells in spleen, respectively. Cytokine secretion was measured by intracellular cytokine staining upon restimulation with overlapping peptide sets spanning the E7 and E6 proteins, respectively. Bars represent the mean±SEM of 5 mice per group. Representative FACS plots are shown in (C). Numbers indicate the percentage of cells falling into the respective quadrants.

Supplementary Figure 4

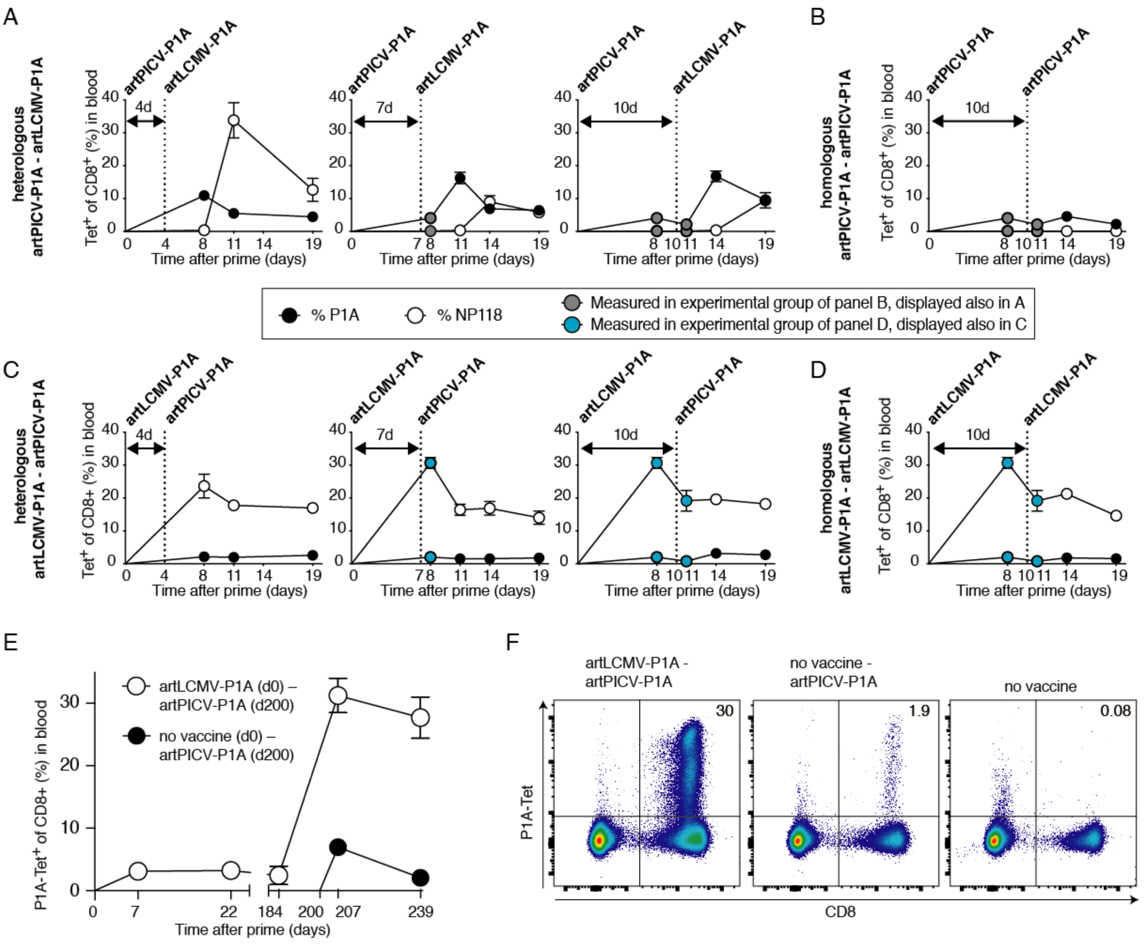

Figure S4: Impact of heterologous artARENA vector prime – boost interval on CTL responses, and efficient artPICV boost of artLCMV-primed responses after a long interval. Related to Figure 3.

A-D: We immunized DBA/2 mice using artLCMV-P1A and artPICV-P1A in heterologous (A,C) or homologous (B,D) prime – boost combinations and at intervals of 4, 7 or 10 days as indicated. P1A-specific and NP118-specific CTL responses in peripheral blood were determined at the indicated time points. Grey and blue symbols are included in the graphs to panels (A,C) for reference only: Grey symbols in (A,B) indicate values determined in the group displayed in (B) or upon single artPICV-P1A prime, blue symbols in (C,D) indicate values determined in the group displayed in (D) or upon single artLCMV-P1A prime. Symbols represent the mean±SEM of n=3-4 mice per group.

E-F: We immunized BALB/c mice with artLCMV-P1A on d0 and left controls without immunization. On d200 both groups were given artPICV-P1A for booster or prime immunization, respectively. P1A-specific $CD8^+$ T cell frequencies in peripheral blood were determined over time. Symbols in (E) represent the mean±SD of n=3 mice per group. Representative FACS plots from d239 are shown in (F), including also a naïve mouse as technical control (“no vaccine”). Values in (F) indicate the percentage of P1A-tetramer-binding cells amongst $CD8^+$ T cells.

Supplementary Figure 5

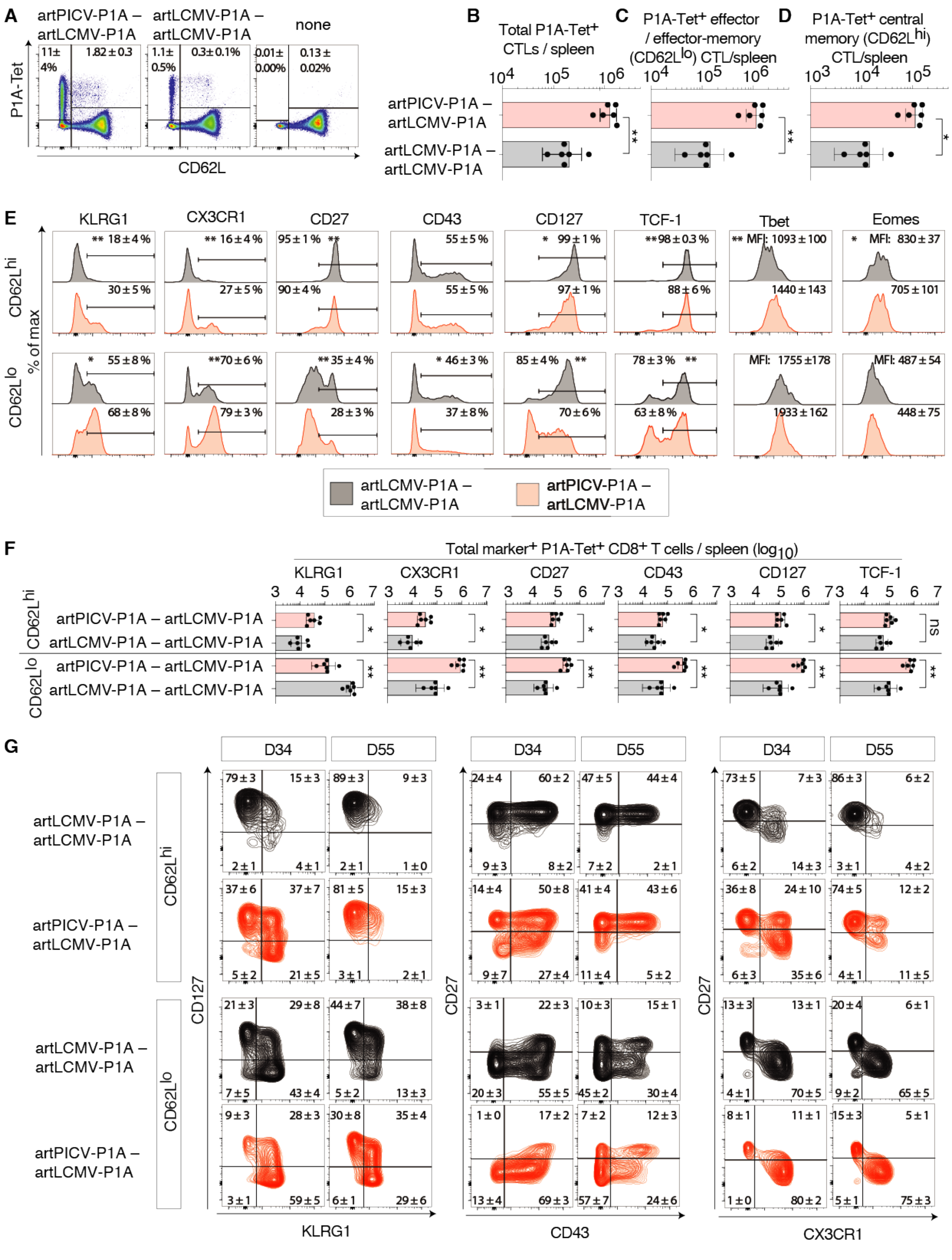

Figure S5: Phenotype of artARENA-induced CTLs one month after boost. Related to Figure 4.

We immunized BALB/c mice with artPICV-P1A and artLCMV-P1A in homologous or heterologous prime – boost vaccination i.v. on d0 and d27. On d34 (G, same experimental data set as shown in Fig. 4A-F) and d55 (A-G) we analyzed P1A-Tet-binding and CD62L expression by splenic CD8^{+} T cells (A, gated on CD8^{+}B220^{-}

lymphocytes). Unimmunized control mice are shown for comparison in (A) only. Numbers in (A) indicate the percentage of cells in the respective quadrant. Total P1A-Tet^{+} CTLs (B), P1A-specific effector/effector-memory CTLs (CD62L^{lo}, C) and P1A-specific central memory CTLs (CD62L^{hi}, D) were enumerated in spleen. In both subsets of P1A-specific CTLs, CD62L^{hi} and CD62L^{lo}, we determined the surface expression of KLRG1, CX3CR1, CD27, CD43 and CD127 as well as the master transcription factors Tcf-1, Tbet and Eomes (E). Total numbers of marker-expressing P1A-specific CTLs were enumerated in (F). Co-expression of CD127, KLRG1, CD27, CD43 and CX3CR1 in combinations as indicated are shown in (G). (A) shows representative FACS plots from individual mice, (G) shows combined events from 6 mice. Symbols in (B-D,F) represent individual mice, bars in (B-D,F) indicate the mean±SD. Numbers in (A,E,G) indicate the percentage of gated cells (mean±SD) or the mean fluorescence intensity (MFI±SD). Means were calculated from six mice per immunization group (A-G) or from three unimmunized controls (A). ** $p<0.01$, * $p<0.05$ by unpaired two-tailed Student's *t* test.

Supplementary figure 6

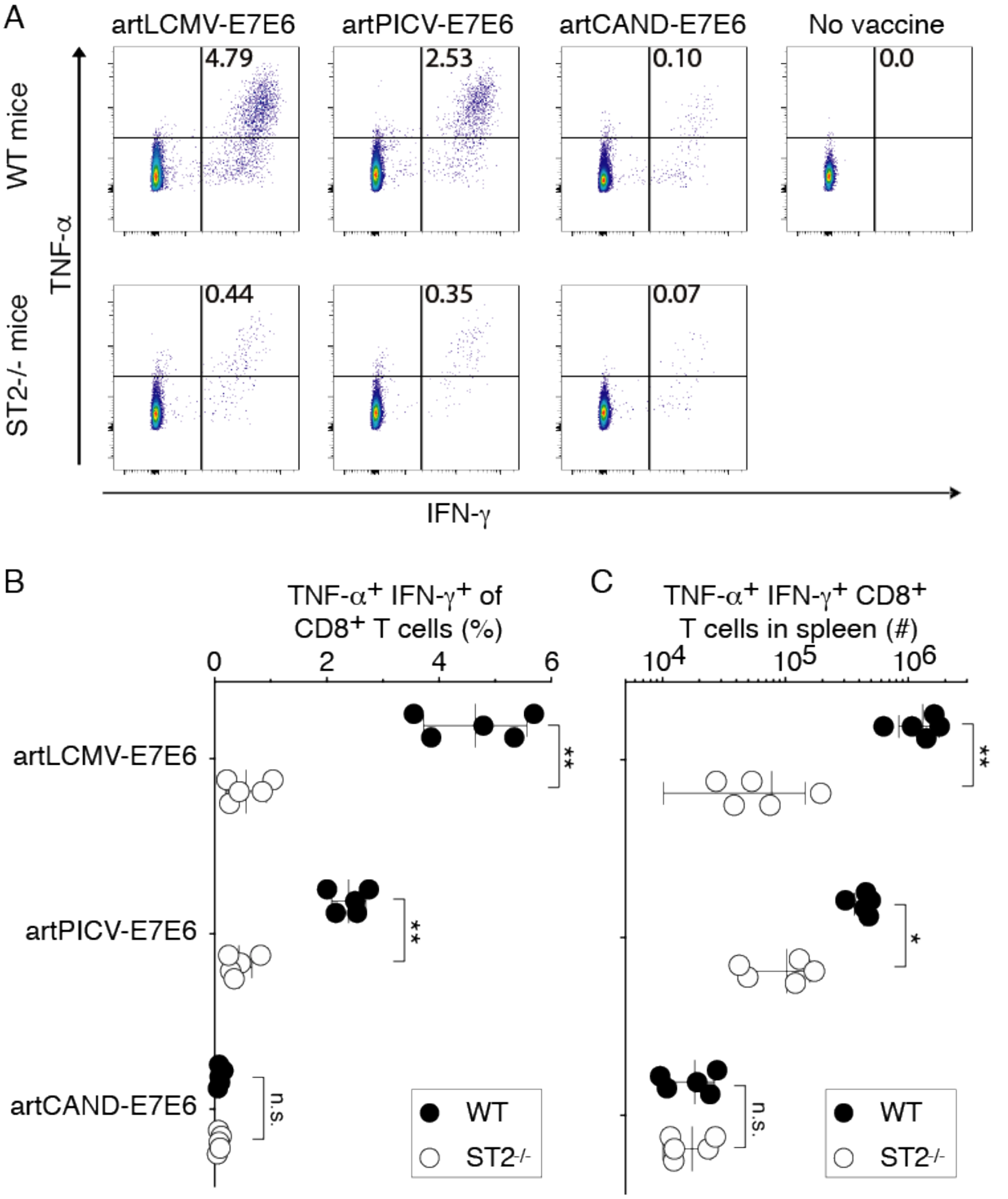

Figure S6: Dependence of artARENA-induced CTL responses on IL-33 – ST2 alarmin signaling. Related to Figure 4.

We immunized ST2-/- and wt mice on d0 with artLCMV-E7E6, artPICV-E7E6 or arCAND-E7E6 i.v. Wt controls were left unimmunized ("no vaccine"). $E7_{49\text{-}57}$ peptide-specific cytokine-secreting cells were determined on d9.

A: Representative FACS plots are shown.

B,C: TNF-α and IFN-γ co-producing $CD8^+$ T cells were enumerated and expressed as percentage of the total splenic $CD8^+$ T cell compartment (B) or as absolute number per spleen (C). Symbols in (B,C) represent individual mice with mean±SD. N=2. ** $p<0.01$ by two-way ANOVA with Sidak's post-test.

Supplementary figure 7

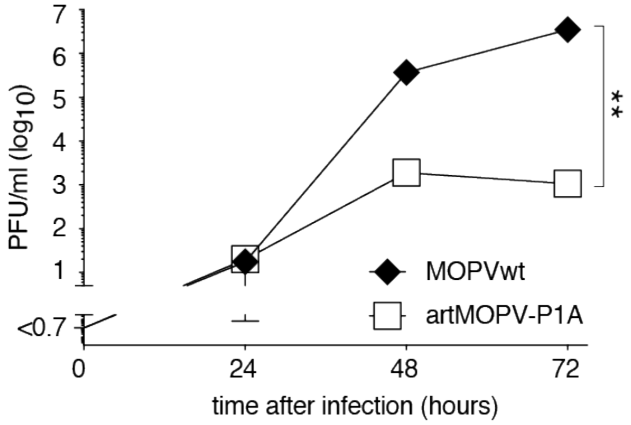

Figure S7: **Cell culture growth curve of artMOPV-P1A. Related to Figure 5.**

We infected BHK-21 cells at MOI=0.01 with artMOPV-P1A or with MOPVwt and determined infectious titers in the supernatant at the indicated time points. Symbols represent the mean±SD of three independent cell culture wells (error bars mostly hidden within the symbol size). ** $p<0.01$ by unpaired two-tailed Student's *t* test.
