# Supplementary material for: Heterologous arenavirus vector prime-boost overrules self-tolerance for efficient tumor-specific CD8 T cell attack
Source: Cell Rep Med. 2021 Mar 3;2(3):100209. doi: 10.1016/j.xcrm.2021.100209 (PMC7974551; doi:10.1016/j.xcrm.2021.100209)
Supplement: Document S2. Article plus supplemental information [file mmc2.pdf]

Cell Reports
Medicine

Article

Heterologous arenavirus vector prime-boost overrules self-tolerance for efficient tumor-specific CD8 T cell attack

Graphical Abstract

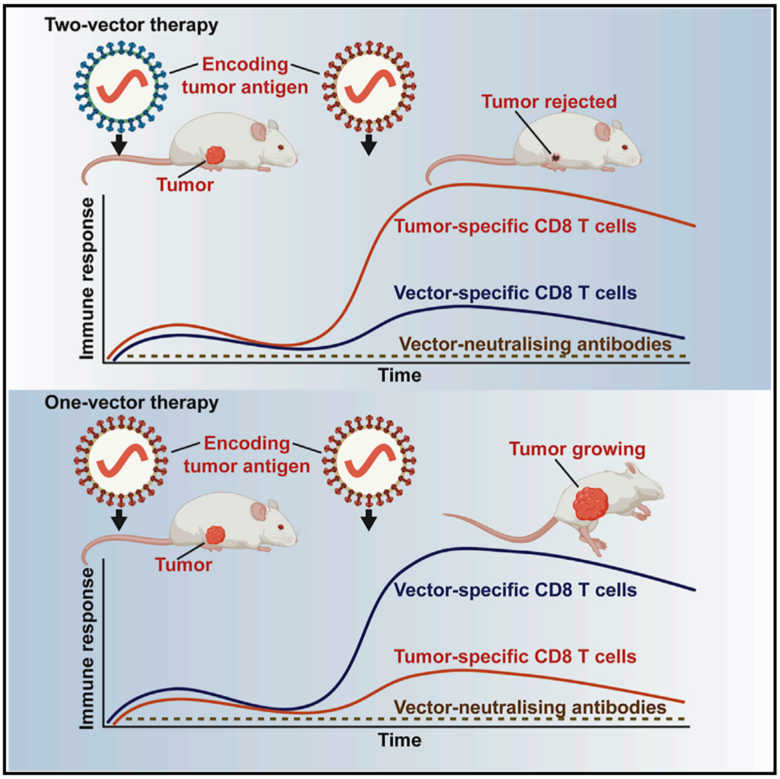

Highlights

- Engineered arenaviruses induce potent tumor self-specific CD8 T cell (CTL) response
- Combinations of distantly but not closely related arenavirus vectors eliminate tumors
- Vector backbone-targeted CTL responses compete against tumor self-reactive CTLs
- Optimized vector combinations reshuffle immunodominance to break self-tolerance

Authors

Weldy V. Bonilla, Nicole Kirchhammer, Anna-Friederike Marx, ..., Klaus K. Orlinger, Alfred Zippelius, Daniel D. Pinschewer

Correspondence

daniel.pinschewer@unibas.ch

In brief

Therapeutic tumor vaccination should break self-tolerance. Assessing combinations of engineered arenavirus vectors, Bonilla et al. find that distantly related vector combinations reshuffle T cell immunodominance hierarchies to break self-tolerance and eliminate established solid tumors, whereas closely related vectors interfere because of immunodominance of anti-vector CD8 T cells rather than antibodies.

Bonilla et al., 2021, Cell Reports Medicine *2*, 100209
March 16, 2021 © 2021 The Author(s).
https://doi.org/10.1016/j.xcrm.2021.100209

CellPress

Cell Reports Medicine

CellPress OPEN ACCESS

Article

Heterologous arenavirus vector prime-boost overrules self-tolerance for efficient tumor-specific CD8 T cell attack

Weldy V. Bonilla,[1] Nicole Kirchhammer,[1,10] Anna-Friederike Marx,[1,10] Sandra M. Kallert,[1] Magdalena A. Krzyzaniak,[1] Min Lu,[1] Stéphanie Darbre,[2] Sarah Schmidt,[3] Josipa Raguz,[3] Ursula Berka,[3] Ilena Vincenti,[2] Mindaugas Pauzuolis,[2] Romy Kerber,[4] Sabine Hoepner,[5] Stephan Günther,[4] Carsten Magnus,[6,9] Doron Merkler,[2,7] Klaus K. Orlinger,[3] Alfred Zippelius,[1,8] and Daniel D. Pinschewer[1,11,*]

[1]University of Basel, Department of Biomedicine, Basel, Switzerland
[2]Department of Pathology and Immunology, University of Geneva, Geneva, Switzerland
[3]Hookipa Pharma Inc., Vienna, Austria
[4]Bernhard Nocht Institute for Tropical Medicine, Hamburg, Germany
[5]Tumor Immunology, Department for BioMedical Research, University of Bern, Bern, Switzerland
[6]Institute of Virology, University of Zurich, Zurich, Switzerland
[7]Division of Clinical Pathology, University Hospitals of Geneva, Geneva, Switzerland
[8]Medical Oncology, University Hospital Basel, Basel, Switzerland
[9]Present address: Diagnostics Information Solution, F. Hoffmann-La Roche AG, Grenzacherstrasse 124, 4070 Basel, Switzerland
[10]These authors contributed equally
[11]Lead contact
*Correspondence: daniel.pinschewer@unibas.ch
https://doi.org/10.1016/j.xcrm.2021.100209

SUMMARY

Therapeutic vaccination regimens inducing clinically effective tumor-specific CD8$^+$ T lymphocyte (CTL) responses are an unmet medical need. We engineer two distantly related arenaviruses, Pichinde virus and lymphocytic choriomeningitis virus, for therapeutic cancer vaccination. In mice, life-replicating vector formats of these two viruses delivering a self-antigen in a heterologous prime-boost regimen induce tumor-specific CTL responses up to 50% of the circulating CD8 T cell pool. This CTL attack eliminates established solid tumors in a significant proportion of animals, accompanied by protection against tumor rechallenge. The magnitude of CTL responses is alarmin driven and requires combining two genealogically distantly related arenaviruses. Vector-neutralizing antibodies do not inhibit booster immunizations by the same vector or by closely related vectors. Rather, CTL immunodominance hierarchies favor vector backbone-targeted responses at the expense of self-reactive CTLs. These findings establish an arenavirus-based immunotherapy regimen that allows reshuffling of immunodominance hierarchies and breaking self-directed tolerance for efficient tumor control.

INTRODUCTION

Cytotoxic CD8$^+$ T lymphocytes (CTLs) are central mediators of adaptive immunity. Tumor-infiltrating CTLs in several tumor types are associated with clinical outcome,[1–3] and pre-existing CTL infiltration may predict responsiveness to immune checkpoint inhibition.[4] Analogously, CTLs are key players in HIV elite control and hepatitis B virus clearance.[5–7]

Therapeutic vaccination for CTL induction holds great promise for cancer therapy[8,9] but has delivered inconsistent therapeutic benefits, including failure of large clinical trials.[10–13] Despite induction of sizeable tumor antigen-specific CD8$^+$ T cell frequencies by modalities such as adjuvanted peptides,[14] inefficient tumor infiltration has curtailed the clinical efficacy of these cells.[15,16] Delivering tumor-associated antigens (TAAs) in the context of virus-induced inflammation[17] has significant potential to overcome these hurdles. Accordingly, several viral vector platforms have been developed for therapeutic use against solid tumors.[13,18–25] The immunostimulatory properties of the viral particles themselves, exhibiting pathogen-associated molecular patterns, activate antigen-presenting cells (APCs) to augment and differentiate immune responses.[26,27] In addition, certain replicating viral delivery systems trigger release of damage-associated molecular patterns or alarmins, such as interleukin-33 (IL-33).[28,29] These signals critically augment activated T cell expansion, effector differentiation, and anti-tumor efficacy.[30,31]

Anti-vector immunity can inhibit viral delivery systems, impeding their re-administration to augment immune responses. A single immunization with vaccinia virus or modified vaccinia virus Ankara (MVA) elicits neutralizing antibody (nAb) responses in the majority of individuals[32] and inhibits vaccinia-vectored immunization.[33] The seroprevalence of common adenovirus (Ad) serotypes such as Ad5 can regionally exceed 90%. Ad5-nAbs

Check for updates

This is an open access article under the CC BY license (http://creativecommons.org/licenses/by/4.0/).

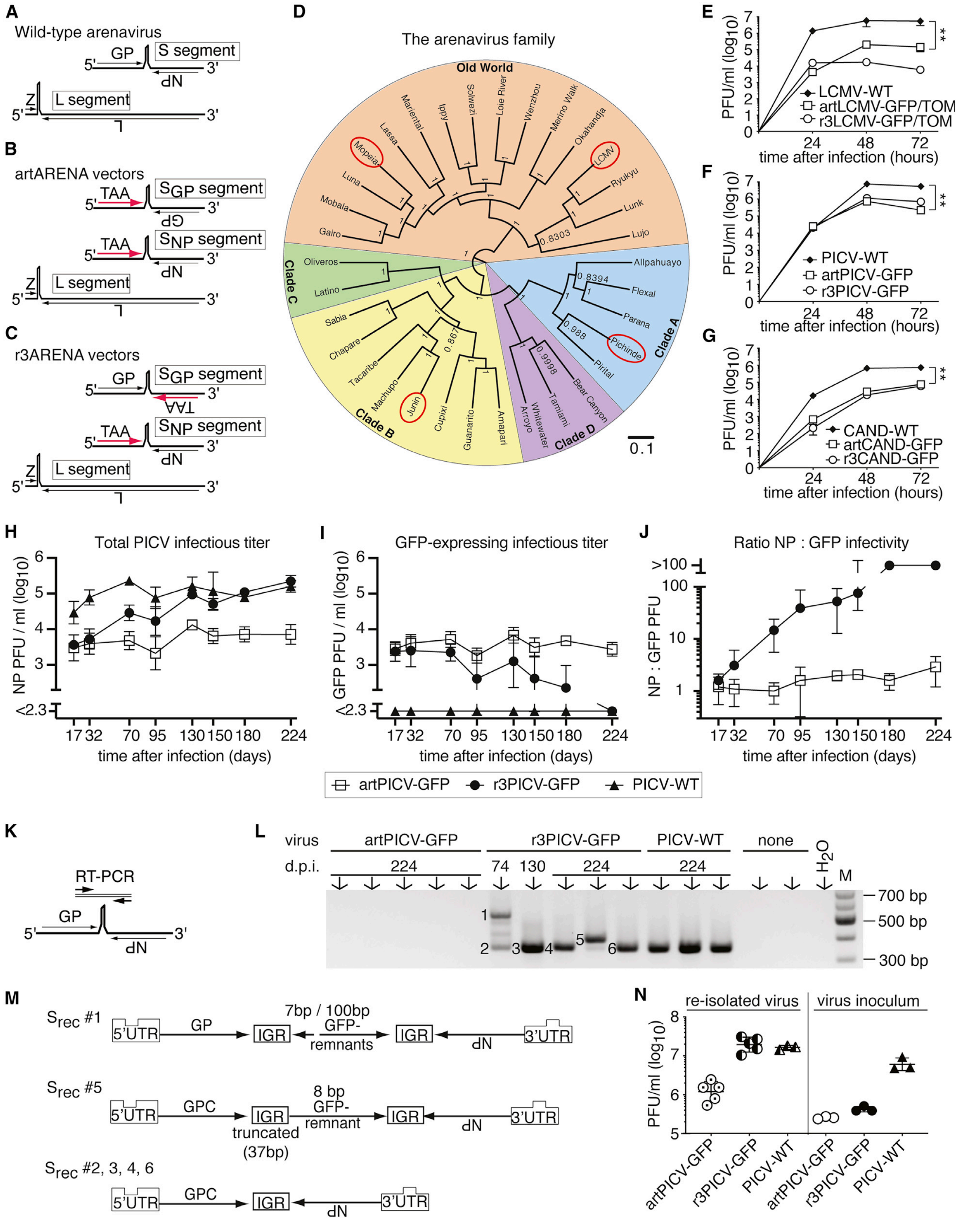

(legend on next page)

dampen or even abrogate responses to recombinant Ad5-based vaccines,[34] and use of simian Ad (sAd) backbones with lower seroprevalence has increased immunological response rates in clinical trials.[35,36] Yet, also sAd vectors induce vector-specific nAbs when used for vaccination and have failed to demonstrate efficient homologous boosting capacity.[37] More surprisingly, even serologically distinct adenovectors did not efficiently boost each other[36,37], and adenoviral vectors are now commonly combined with poxvirus-based platforms for heterologous prime-boost vaccination.[37]

For several decades, members of the Arenaviridae family have found widespread use in basic immunological research because of their capacity to induce CTL responses of exceptional magnitude, functionality, and longevity.[38] Reverse genetic techniques have enabled the tailored design of this virus family and its exploitation for vaccination.[39,40] Replication-deficient vectors (rLCMV) based on the prototypic arenavirus lymphocytic choriomeningitis virus (LCMV) have demonstrated excellent CTL and nAb induction against vectorized transgenes in mice, non-human primates, and, recently, also in humans.[41–44] As an important differentiation from other viral vector technologies, rLCMV-based immunization only rarely induces vector-nAbs, facilitating the vector's repeated administration in homologous prime-boost vaccination.[41,43,44] This peculiarity of LCMV-based vectors is due to an N-linked "glycan shield" on the outer globular domain of the viral envelope glycoprotein domain that impairs antibody accessibility to critical neutralizing epitopes.[45] rLCMV vectors are currently in clinical phase 2 testing for prevention of cytomegalovirus-associated disease in transplant recipients.[46]

We and others have developed replication-attenuated, trisegmented, arenavirus-based vector formats (artARENA, r3ARENA; Figures 1B and 1C) that induce even more potent effector CTL responses than replication-deficient rLCMV or commonly used poxvirus and Ad vector systems.[29,47,48] An LCMV-based artARENA vector (artLCMV) has recently entered early-stage clinical testing.[49] When used to deliver tumor self-antigens to tumor-bearing mice, artLCMV induced potent antitumor CTL responses and extended the animals' survival.[29] Tumor-specific CTL responses were, however, lower in magnitude than those induced against vectorized non-self-antigens, and complete tumor remission was not achieved. This suggested that self-tolerance limited the therapeutic efficacy of artLCMV-based immunization.[29]

Here we systematically explore a range of additional arenavirus vector backbones for vaccine delivery. We report that, contrary to commonly held concepts, anti-vector CTL responses rather than nAbs curtail the immunogenicity of homologous arenavirus vector prime-boost vaccination. Accordingly, backbones of distant genealogic relationship offered the most potent heterologous prime-boost combinations, resulting in considerable rates of complete tumor rejection.

RESULTS

Arenavirus vector backbone candidates and their genetic and phenotypic stability

Arenaviruses form enveloped particles and contain two segments of negative-stranded RNA. The large (L) segment encodes for the viral polymerase L and the matrix protein Z, whereas the short (S) segment carries the envelope glycoprotein (GP) and nucleoprotein (NP) genes, separated by intergenic regions, respectively (Figure 1A). We and others have incorporated transgenic sequences such as TAAs into replicating arenavirus vectors by segregating the NP and GP genes onto artificially duplicated S segments (S_{NP} and S_{GP}; Figures 1B and S1C).[29,47,48] This can be achieved by the artARENA (e.g., artLCMV) or r3ARENA (e.g., r3LCMV) design strategy (Figures 1B and 1C). Here we vectorized additional mammalian arenaviruses (mammarenaviruses) to exploit their immunotherapeutic potential when combined in heterologous prime-boost combinations. Based on phylogenetic relationship, mammarenaviruses can be subdivided into the Old World group of viruses and four clades (A–D) of New World viruses (Figure 1D). For vector generation (STAR methods), we selected three mutually very distantly related viruses representing the main branches of the phylogenetic tree: the prototypic Old World virus LCMV, the widely studied New World clade A virus Pichinde virus (PICV), and the Junin virus vaccine strain Candid#1 (CAND). CAND is in clinical use as a prophylactic vaccine against Junin virus, the causative agent of Argentine hemorrhagic fever,[50] and PICV has no human disease correlate but can infect humans, as documented in accidentally exposed laboratory workers.[51] LCMV infection is mostly asymptomatic or manifests as a flu-like infection.[52] Rare cases of choriomeningitis are generally self-limiting and, despite documented cases of protracted central nervous system manifestations, commonly heal without persisting sequelae.[53,54] From a safety

Figure 1. Arenavirus vector backbone candidates and their genetic and phenotypic stability

(A–C) Schematic of the genome organization of WT arenaviruses (A), artARENA vectors (B), and r3ARENA vectors (C). TAA, tumor-associated antigen.

(D) Genealogy tree of the mammarenavirus family with its clades. Red circles indicate viruses used in this study. The scale bar describes the expected number of mutations per site.

(E–G) Growth curves of the indicated viruses and vectors in BHK-21 cells (E and F) and 293T cells (G) infected at a multiplicity of infection (MOI) of 0.01. Symbols show the mean ± SD of three cell culture wells (error bars mostly within symbol size). **$p < 0.01$ by unpaired two-tailed Student's t test.

(H–J) AGRAG mice were infected intravenously (i.v.) with the indicated viruses, and viremia was monitored by immunofocus assays detecting PICV-NP (H) or GFP (I) to calculate the NP:GFP infectivity ratio (J). Symbols represent means ± SD of 3–5 mice.

(K–M) RT-PCR strategy (K) to amplify recombined WT-like PICV S segment RNA species re-uniting NP and GP sequences. Also shown is a gel electrophoresis image of RT-PCR products (L) obtained from serum samples collected at the indicated time points. Each lane represents an individual mouse. Sera from uninfected mice ("none") and water were included as negative controls. Sequence analysis of the bands numbered in (L) suggested recombination products, as depicted schematically in (M). IGR, S segment intergenic region; UTR, untranslated region.

(N) BHK-21 cells were infected with viruses re-isolated from individual AGRAG mice on day 224 of the experiment shown in (H)–(J) or with the viral stocks originally used to infect the animals. Titers after 72 h are shown. Symbols represent individual virus cultures from one mouse each. Mean ± SD is indicated.

Number of independent datasets (N) for (H)–(N) = 2. See also Figure S1.

perspective, all three viruses are valid vector backbone candidates for immunotherapy use in humans. We generated artARENA (artLCMV, artPICV, and artCAND) as well as r3ARENA (r3LCMV, r3PICV, and r3CAND) vectors expressing fluorescent reporter proteins. In cell culture, artARENA vectors as well as their respective r3ARENA counterparts reached lower titers than their respective parental wild-type (WT) viruses (Figures 1E–1G). These observations extended and generalized earlier findings of attenuated artARENA and r3ARENA vector growth.[29,47,48]

Genetic and phenotypic stability are key criteria for the clinical utility of replicating viral vector systems. We have reported previously that artLCMV stably retained its genome organization and transgene expression over extended periods of *in vivo* replication, whereas r3LCMV underwent inter-segmental recombination, reverting to a non-transgenic bisegmented WT-like virus.[29] Here we used artPICV-GFP, r3PICV-GFP, and WT PICV (PICV-WT) to infect highly immunodeficient mice, which lack type I and type II interferon receptors and are devoid of T and B cells because of RAG deficiency (AGRAG mice). In the first 30 days of persistent infection, total viral loads (determined as PICV NP-expressing infectivity) in the blood of artPICV-GFP- and r3PICV-GFP-infected animals were similar and below those of PICV-WT-infected controls (Figure 1H). By day 70, however, r3PICV-GFP viremia exceeded the levels in artPICV-GFP-infected animals and increased continuously thereafter, eventually reaching levels equivalent to PICV-WT. Conversely, artPICV viremia remained consistently below PICV-WT controls. GFP transgene-expressing viral infectivity in r3PICV-GFP- and artPICV-GFP-infected mice was at comparable levels up to around day 70 of infection (Figure 1I). Thereafter it declined continuously in r3PICV-GFP-infected animals but remained stable in artPICV-infected animals. The resulting ratio of total PICV infectivity to GFP-transgenic infectivity documented that r3PICV-GFP progressively lost its transgene, whereas transgene expression by artPICV-GFP remained stable throughout the observation period of more than 200 days (Figure 1J). We performed RT-PCR to detect supposedly recombined WT-like S segments containing PICV NP and GP sequences (Figure 1K). Such RNA species were absent from artPICV-GFP-infected mice but detected consistently in the blood of r3PICV-infected animals (Figure 1L). Sequence analysis of amplicons revealed that some of them contained one or two GFP remnants flanked by partially or completely duplicated viral intergenic regions, identifying them as inter-segmental recombination products of the S_{NP} and S_{GP} segments of r3PICV-GFP (Figure 1M). On day 224 after infection, RT-PCR assays detected the S_{NP} and S_{GP} segments of artPICV-GFP but not the corresponding GFP-containing segments of r3PICV-GFP (Figures S1A–S1D), further supporting the notion of r3PICV-GFP transgene loss. When re-isolated after more than 200 days of persistent infection and propagated in cell culture, r3PICV-GFP reached titers equivalent to PICV-WT, whereas re-isolated artPICV-GFP growth was attenuated. This contrasted with the cell culture behavior of the inoculum of r3PICV-GFP and artPICV-GFP, both of which grew to lower titers than the corresponding PICV-WT (Figure 1N). These findings indicated that the genetic instability of r3PICV-GFP was accompanied by phenotypic reversion to PICV-WT-like growth, whereas artPICV-GFP was genetically and phenotypically stable. Analogous findings were made with CAND-based vectors. S_{NP}-S_{GP} recombination products and loss of GFP-containing segments was also observed in r3CAND-infected AGRAG mice but not in artCAND-infected animals (Figures S1E–S1K). Re-isolated r3CAND-GFP exhibited CAND-WT-like cell culture growth behavior (Figure S1L), whereas artCAND replication in AGRAG mice was at levels too low to allow virus re-isolation. These studies generalized the finding[29] that artARENA vectors are genetically and phenotypically stable, whereas r3ARENA vectors are prone to transgene loss and phenotypic reversion to a WT-like virus.

artARENA vectors are attenuated in guinea pig and mouse pathogenesis models

Next we tested whether artPICV and artLCMV were attenuated in animal models. Guinea pigs were infected with titrated doses of PICV-WT, known to cause lethal disease in these animals, or with artPICV-E7E6 expressing a non-oncogenic fusion construct consisting of the HPV16 E7 and E6 open reading frames (ORFs)[55] (see chart in Figure 2A). Animals receiving diluent were included as a further control. At PICV-WT doses of 3 × 10e2 or 3 plaque-forming units (PFUs), three of eight animals reached humane endpoints (Figure 2A), a disease that was always accompanied by high-level viremia (>10e3 PFUs; Figures 2B and 2C). At the highest PICV-WT dose (3 × 10e4 PFUs), seven of eight guinea pigs developed high-level viremia and terminal disease (Figures 2A and 2D). In contrast, artPICV-E7E6 infection at 3 × 10e2 PFUs was aviremic (Figure 2E), and doses of 3 × 10e4 or even 3 × 10e6 PFUs (100-fold higher than the highest PICV-WT dose tested) did not result in high-level viremia (Figures 2F and 2G). Transient low-level viremia was detected in only 1 of 8 animals in both of these latter cohorts (Figures 2F and 2G), and none of these animals developed terminal disease (Figure 2A). When administered 3 × 10e2 PFUs of artPICV-E7E6, the lowest dose tested, seven of eight animals were free of disease throughout the 27-day observation period, and none of the animals had detectable viremia (Figure 2E). On day 24 after vector inoculation, one of these aviremic animals suddenly exhibited signs of disease corresponding to humane study endpoints, necessitating its euthanasia on day 25. The absence of detectable viral loads in this animal suggested, however, that its disease was unrelated to artPICV-E7E6 vector administration. Measurements of body weight loss, a commonly used parameter of PICV-induced disease in guinea pigs, provided additional independent support for the conclusion that artPICV-E7E6 was substantially attenuated (Figure S2).

Intracranial inoculation of mice is the standard model to assess the neurovirulence of LCMV-based vectors.[29,41,56] In agreement with earlier data demonstrating attenuation of artLCMV vectors,[29] we found that 100–1,000 PFUs of artLCMV-E7E6 had to be administered intracranially to elicit terminal choriomeningitis in about half of the animals, whereas 1 PFU of LCMV-WT resulted in terminal disease in all mice (Figure 2I). LCMV-induced choriomeningitis is mediated by CD8 T cells, which attack virus-infected meningeal cells and

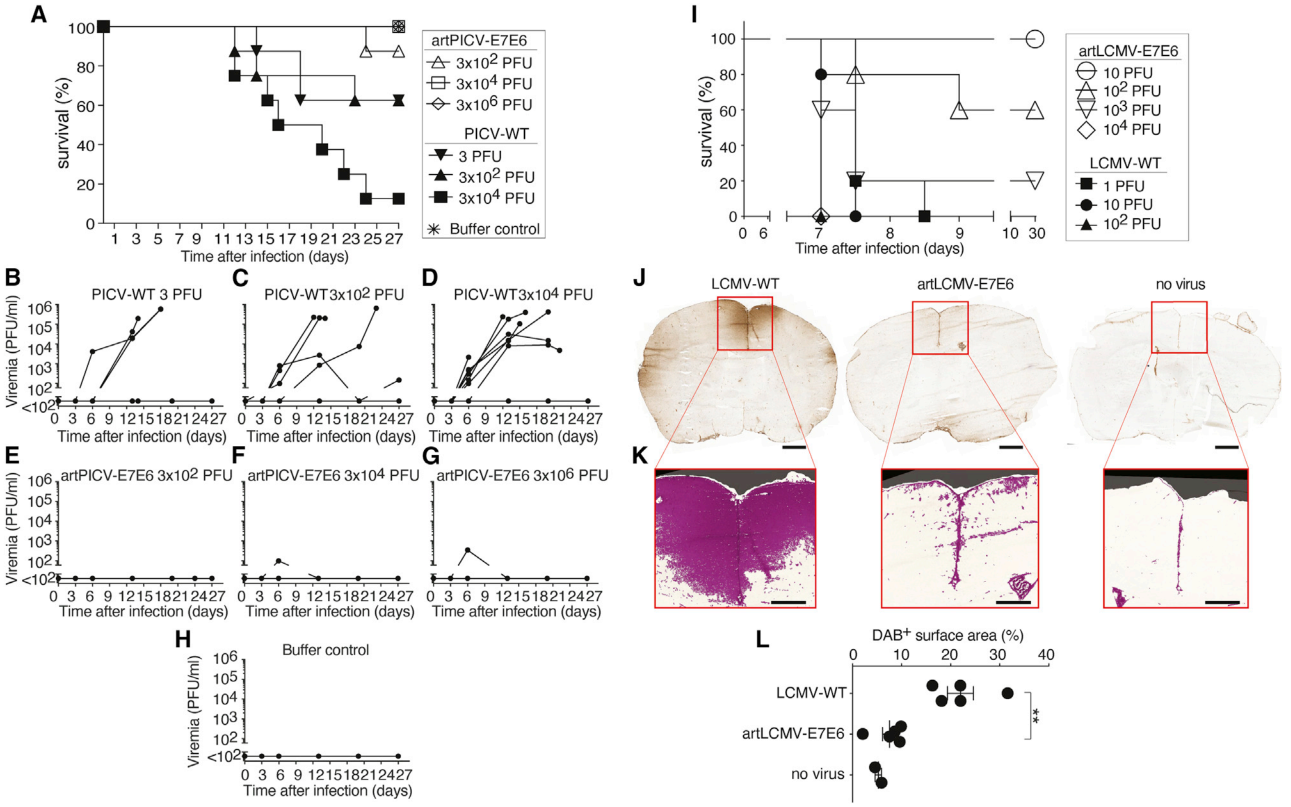

Figure 2. artARENA vectors are attenuated in guinea pig and mouse pathogenesis models

(A–H) We intraperitoneally infected groups of 8 adult Hartley guinea pigs, four of each sex, with artPICV-E7E6 or PICV-WT at the indicated doses and monitored humane endpoints ("survival"). A group of six control animals (three of each sex) was administered diluent.

(A) The animals were monitored until reaching humane endpoints or until the end of the study on day 27.

(B–H) Viral loads in the blood of the same eight or six animals per group, respectively, as shown in (A), were determined by immunofocus assay.

(I) We inoculated groups of five C57BL/6 mice intracranially with titrated doses of LCMV-WT or artLCMV-E7E6 as indicated and monitored them for signs of choriomeningitis ("survival").

(J–L) To analyze the effect of viral infection on BBB permeability, animals were inoculated intracranially with 10 PFUs of LCMV-WT, 10 PFUs of artLCMV-E7E6, or with diluent. Seven days later, immunoglobulin G (IgG) in the brain parenchyma, indicating leakage across the BBB, was detected by peroxidase-based immunohistochemistry on histological sections. Representative images of coronary brain sections are shown (J), with an enlargement of computer-assisted detection of the IgG-positive surface (K). Quantitation of the detected area is shown in (L). Error bars in (L) show the mean ± SEM, and dots represent individual mice. Scale bars show 1,000 μm (J) and 500 μm (K).

See also Figure S2.

astrocytes, resulting in blood-brain barrier (BBB) breakdown and brain edema.[57–59] To investigate the mechanisms underlying reduced artLCMV neurovirulence, we infected WT mice intracranially with 10 PFUs of artLCMV-E7E6 or LCMV-WT or with diluent. Seven days later, at the peak of disease in LCMV-WT-infected animals, we determined immunoglobulin deposits in the brain parenchyma as a surrogate of BBB integrity (Figures 2J and 2K). The brain area affected by BBB breakdown was significantly larger in LCMV-WT- than in artLCMV-E7E6-infected mice (Figure 2L). Regions of dense immunoglobulin deposits were evident in LCMV-WT-infected brains, notably around the longitudinal fissure, with substantial extension into deeper cortical layers, whereas in artLCMV-E7E6-infected brains, only small immunoglobulin deposits were detected in proximity to the longitudinal fissure. This morphological correlate of reduced immunopathological damage furthers our understanding of attenuated artLCMV neurovirulence.[57]

Immunogenicity and epitope dominance in heterologous artARENA prime-boost vaccination

Next we tested the utility of artPICV-artLCMV as a heterologous prime-boost regimen. We primed C57BL/6 mice with artLCMV-E7E6 or artPICV-E7E6, followed by artLCMV-E7E6 boost on day 13. E7-specific CTL responses in the blood on day 9 were somewhat higher upon artLCMV-E7E6 prime than after artPICV-E7E6 prime (Figure 3A). Seven days after heterologous artLCMV-E7E6 boost (day 20), artPICV-E7E6-primed animals reached E7-specific CTL frequencies of more than 50% of the total circulating $CD8^+$ T cell pool, with only limited contraction over a 1-month-period (Figures 3A–3C and S3). These CTL frequencies vastly exceeded those induced by homologous artLCMV-E7E6 prime-boost, which remained in the 6%–7% range, similar to the frequencies after prime. All of these responses were polyfunctional, as determined by interferon γ (IFN-γ), tumor necrosis factor alpha (TNF-α), and IL-2 secretion

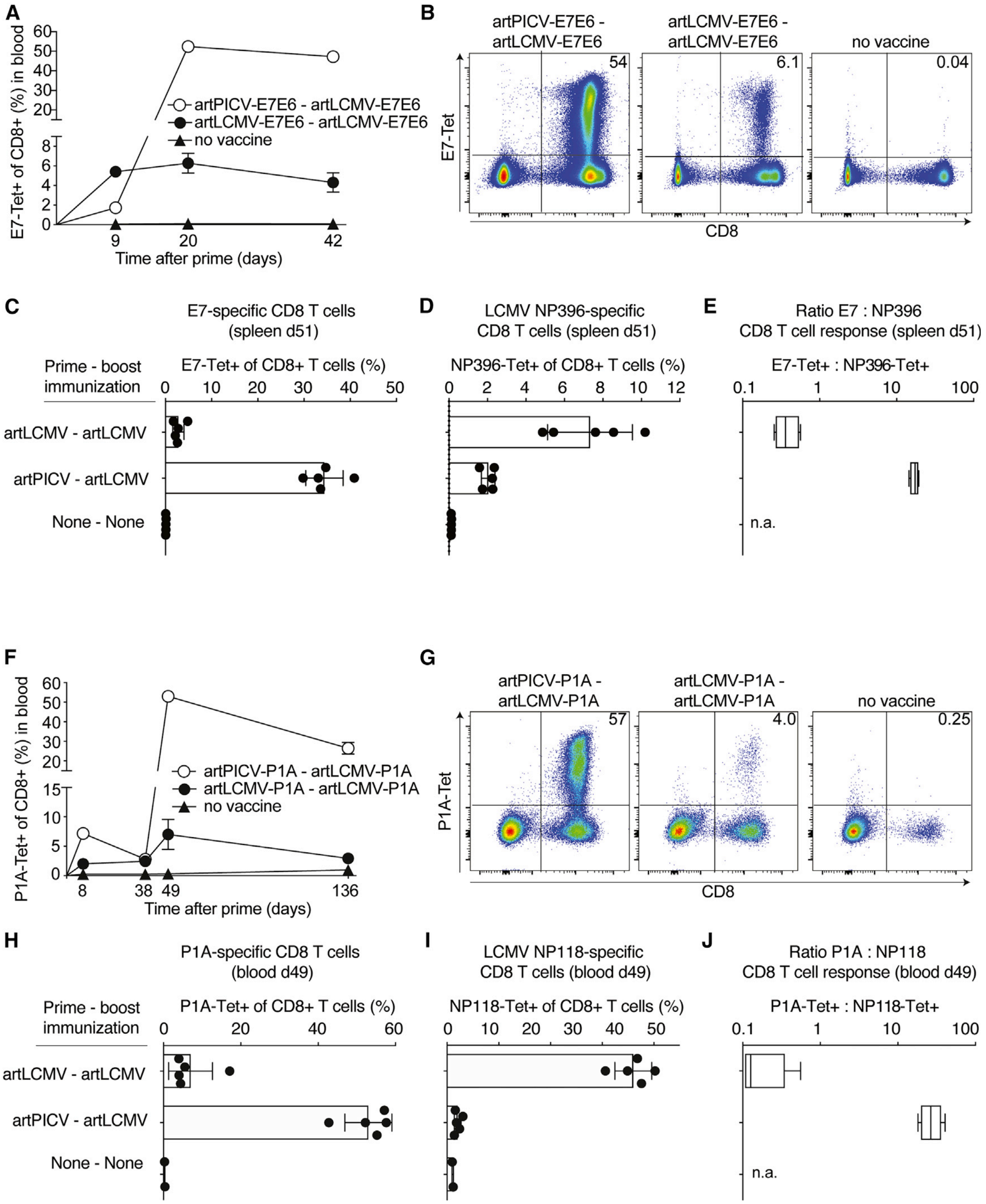

Figure 3. Immunogenicity and epitope dominance in heterologous artARENA prime-boost vaccination

(A–E) C57BL/6 mice were given artPICV-E7E6 and artLCMV-E7E6 homologous or heterologous prime-boost vaccination i.v. on day 0 and day 13. E7-tetramer-binding $CD8^+$ T cell frequencies in the blood were determined at the indicated time points (A). Also shown are representative fluorescence-activated cell sorting (FACS) plots from blood gated on $B220^-$ lymphocytes analyzed on day 20 (B). Splenic frequencies of E7-specific (C) or NP396-specific (D) $CD8^+$ T cells on day 51 served to calculate the epitope dominance ratio displayed in (E).

(F–J) BALB/c mice were given artPICV-P1A and artLCMV-P1A homologous or heterologous prime-boost vaccination i.v. on day 0 and day 39. P1A-tetramer-binding $CD8^+$ T cell frequencies in the blood were determined at the indicated time points (F). Also shown are representative FACS plots from blood gated on $B220^-$ lymphocytes on day 49 (G). Frequencies of P1A-specific (H) or NP118-specific (I) $CD8^+$ T cells in the blood on day 49 served to calculate epitope dominance ratios as displayed in (J).

Symbols in (A) and (F) represent the mean +/– SEM of five mice, except the “no vaccine” groups: 4 mice in (A) and 2 mice in (F). Symbols in (C), (D), (H), and (I) represent individual mice, and bars indicate the mean ± SD. Boxes in (E) and (J) display the minimal and maximal values. Numbers in (B) and (G) indicate the percentage of tetramer-binding cells among $CD8^+B220^-$ T cells. See also Figures S3 and S4.

CellPress
OPEN ACCESS

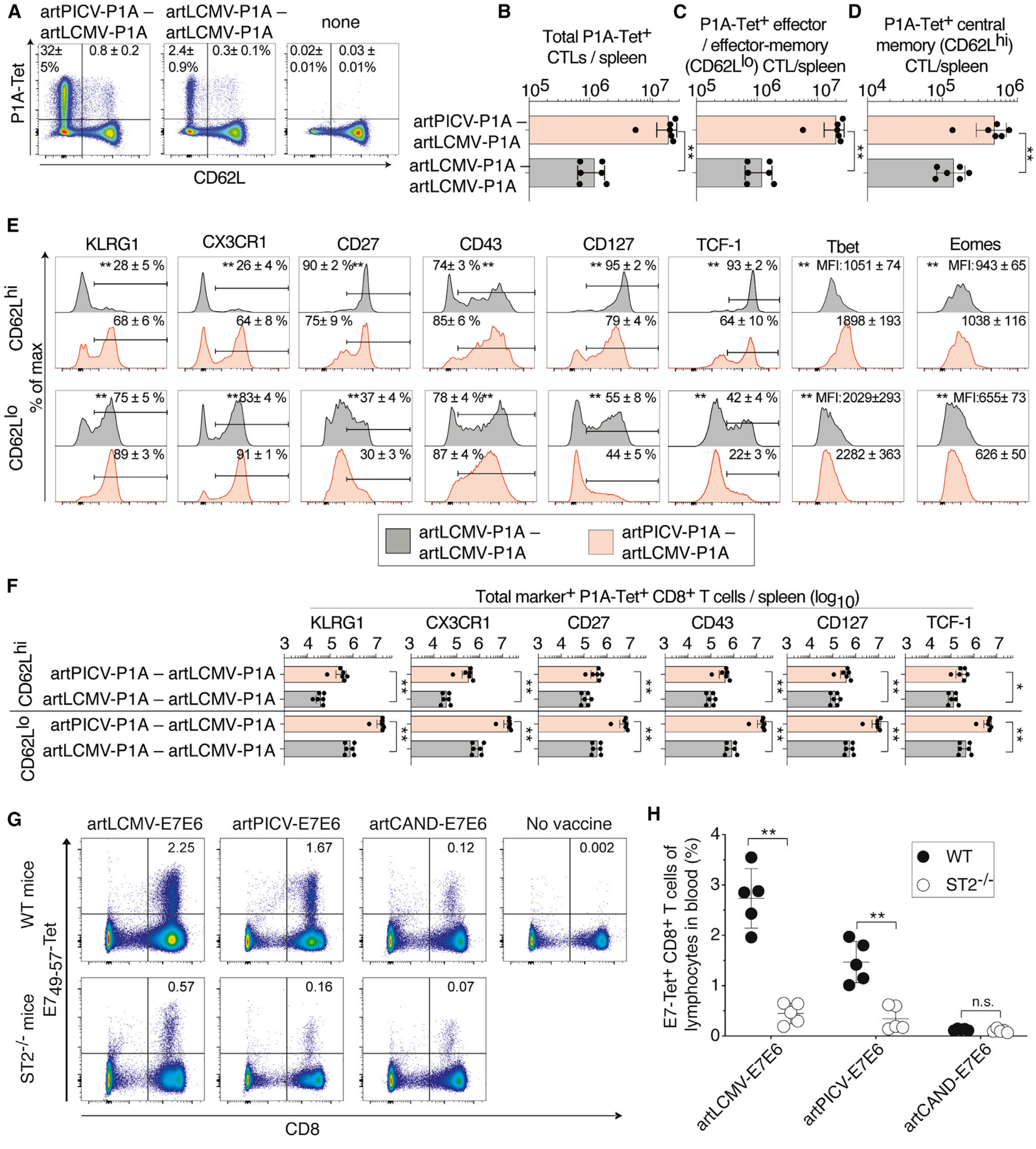

Figure 4. Phenotype of artARENA-induced CTLs and their dependence on IL-33-ST2 alarmin signaling

(A–F) We immunized BALB/c mice with artPICV-P1A and artLCMV-P1A in homologous or heterologous prime-boost vaccination i.v. on day 0 and day 27. On day 34, we analyzed P1A-Tet-binding and CD62L expression by splenic $CD8^+$ T cells (A; gated on $CD8^+B220^-$ lymphocytes). Unimmunized control mice are shown for comparison in (A) only. Numbers in (A) indicate the percentage of cells in the respective quadrant. Total P1A-Tet^+ CTLs (B), P1A-specific effector/effector memory CTLs ($CD62L^{lo}$; C), and P1A-specific central memory CTLs ($CD62L^{hi}$; D) were enumerated in the spleen on day 34. In both subsets of P1A-specific CTLs, $CD62L^{hi}$ and $CD62L^{lo}$, we determined the surface expression of KLRG1, CX3CR1, CD27, CD43, and CD127 as well as the master transcription factors Tcf-1, Tbet, and Eomes (E). Total numbers of marker-expressing P1A-specific CTLs were enumerated in (F). (A) shows representative FACS plots from individual mice. Symbols in (B)–(D) and (F) represent individual mice, and bars in (B)–(D) and (F) indicate the mean ± SD. Numbers in (A) and (E) indicate the percentage of gated cells (mean ±

(legend continued on next page)

upon peptide stimulation and comprised E7- as well as E6-specific CTLs (Figure S3). The majority of tumors, however, do not exhibit viral determinants such HPV E7 and E6, and therefore active immunization has to rely on other classes of TAAs for which immune responses can be affected by self-tolerance. Studies of the P815 mouse tumor cell-derived cancer-testis antigen P1A, for example, have shown that post-natal expression is restricted to spermatogonia, placenta, and thymic medullary epithelial cells, with the latter being key for central tolerance induction.[60,61] Accordingly, P1A knockout mice spontaneously rejected P815 tumors, mounting P1A-specific CD8 T cell responses of significantly higher magnitude and functional avidity than WT animals.[62] To test whether heterologous artARENA immunization facilitates breaking self-tolerance, we immunized BALB/c mice with P1A-expressing artLCMV-P1A or artPICV-P1A. By day 38 after prime, the P1A-specific responses induced by either vector had leveled off in the range of 2%–3% of circulating $CD8^+$ T cells (Figure 3F). Upon heterologous artLCMV-P1A boost, artPICV-P1A-primed mice mounted P1A-specific CTL responses exceeding 50% of the circulating $CD8^+$ T cell pool, substantially higher than upon artLCMV-P1A homologous boost (Figures 3F–3H). These heterologous prime-boost-induced CTL responses contracted slowly, with frequencies of more than 20% persisting in peripheral blood for more than 3 months (Figure 3F). Besides vector transgene-specific responses (E7E6 and P1A), we also determined dominant vector backbone-specific responses directed against the NP-derived epitopes NP396 in C57BL/6 mice (H-$2D^b$ restricted) and NP118 in BALB/c mice (H-$2L^d$ restricted). Interestingly, the responses of C57BL/6 and BALB/c mice, respectively, were substantially lower in animals receiving heterologous artPICV-artLCMV immunization compared with homologous artLCMV prime-boost (Figures 3D and 3I). Based on these measurements we calculated the transgene:backbone epitope dominance ratio (E7:NP396 [Figure 3E]; P1A:NP118 [Figure 3J]). It was substantially higher in heterologous compared with homologous prime-boost immunization (~50-fold in Figure 3E; >200-fold in Figure 3J), indicating that heterologous prime-boost immunization biased vaccine-induced CTL responses toward transgene-derived epitopes. In the context of active immunization for immunotherapy, it is important to induce high-frequency CTL responses within a short time window. artPICV-P1A-artLCMV-P1A heterologous prime-boost, administered at an interval of only 4–10 days, induced P1A-specific CTL responses of higher frequencies than obtained with homologous prime-boost immunization given at the same interval (Figures S4A–S4D). A trend of higher P1A-specific CTL frequencies at longer prime-boost intervals (7–10 days compared with 4 days) was accompanied by an inverse trend in NP118 backbone-targeting responses. artPICV-P1A prime followed by artLCMV-P1A boost clearly outperformed the inverse sequence of administration when administered at intervals of 4–10 days (Figures S4A–S4D). The prime-boost regimen of artPICV followed by artLCMV rather than the inverse sequence of administration was therefore used for subsequent studies. Only when the interval between the two vaccinations was substantially longer was the artPICV-P1A boost of artLCMV-P1A-primed responses effective (Figures S4E and S4F).

Phenotype of artARENA-induced CTLs and their dependence on IL-33-ST2 alarmin signaling

Next, we determined how heterologous artPICV-artLCMV prime-boost influenced the magnitude and phenotype of P1A-specific CTL populations. Seven days after boost, $CD62L^{lo}$ effector/effector memory cells dominated the responses of BALB/c mice to homologous artLCMV-P1A prime-boost and heterologous artPICV-P1A-artLCMV-P1A immunization (Figures 4A–4D). Importantly, heterologous prime-boost elicited not only higher P1A-specific $CD62L^{lo}$ effector/effector memory CTL populations than homologous prime-boost but the $CD62L^{hi}$ central memory population was also more abundant (Figures 4C and 4D). We studied the phenotype of both CTL subsets by determining the cells' expression of the surface markers KLRG1, CX3CR1, CD27, CD43, and CD127 and of the transcription factors Tcf-1, Tbet, and Eomes (Figures 4E and S5G). Within the $CD62L^{hi}$ and $CD62L^{lo}$ subsets of CTLs, heterologous prime-boost immunization promoted expression of the effector differentiation markers KLRG1, CX3CR1, and CD43,[63–67] with a concomitant reduction in the proportion of cells expressing the memory markers CD27 and CD127.[66,68] We also observed that CTLs emerging from heterologous prime-boost expressed higher average Tbet levels and lower levels of Tcf-1, further supporting the conclusion that heterologous prime-boost augmented the effector differentiation of CTLs. Irrespective of this relative effector differentiation bias, heterologous prime-boost augmented not only the total number of CTLs expressing the effector differentiation markers KLRG1, CX3CR1, and CD43 but also the population of cells expressing memory precursor markers (CD27 and CD127) and the stemness-defining transcription factor Tcf-1 (Figure 4F).[69] When analyzed 4 weeks after boost (Figures S5A–S5F), most of the differences between CTLs emerging from homologous and heterologous prime-boost persisted but, overall, were less pronounced than on day 7 after boost. Prominent populations of effector-like memory populations, characterized by the marker combinations $CD43^-CD27^-$, $CX3CR1^+CD27^-$, and $KLRG1^+CD27^-$,[63–67] were particularly abundant upon heterologous prime-boost immunization (Figure S5G). These observations indicated that CTL responses induced by heterologous prime-boost differed from those emerging from homologous prime-boost primarily by their higher cellularity paired with more pronounced effector differentiation.

SD) or the mean fluorescence intensity (MFI ± SD). Means were calculated from six mice per immunization group (A–F) or from three unimmunized controls (A). N = 2. **p < 0.01 by unpaired two-tailed Student's t test.
(G and H) We immunized *ST2*$^{-/-}$ and WT mice with artLCMV-E7E6, artPICV-E7E6 or arCAND-E7E6 i.v. Controls were left unimmunized ("no vaccine" in G). E7-tetramer-binding cells in blood were determined on day 7. Representative FACS plots are shown in (G); values indicate E7-Tet^+CD8^+ T cells as a percentage of lymphocytes. Symbols in (H) represent individual mice (n = 5 per group) with mean ± SD. N = 2. **p < 0.01 by two-way ANOVA with Sidak's post-test.
See also Figures S5 and S6.

CellPress
OPEN ACCESS

IL-33 signals through its receptor ST2 are key for protective CTL responses to replicating viruses,[31] and IL-33 signals critically augment CTL responses to artLCMV-vectored immunization and the resulting tumor control.[29] Here, we compared the ST2 dependence of artARENA-induced CTL responses by immunizing ST2-deficient and WT control mice with artLCMV-E7E6, artPICV-E7E6, or artCAND-E7E6. artLCMV- and artPICV-induced CTL responses to E7 were significantly lower in the blood of ST2-deficient mice than in WT controls (Figures 4G and 4H). Conversely, ST2 deficiency had no clear effect on artCAND-induced CTL responses. ST2-dependent differences in artLCMV- and artPICV-induced CTL responses were also evident when enumerating IFN-γ and TNF-α co-producing CTLs in the spleen (Figure S6). These findings indicated that, besides artLCMV, artPICV- but not artCAND-induced CTL responses also benefitted from IL-33 signaling.

Genealogic artARENA vector backbone relatedness dictates interference by pre-existing immunity and potency in heterologous prime-boost immunization

Our observations shown in Figures 3E and 3J suggested that heterologous artARENA prime-boost immunization overruled the immunodominance of vector backbone-directed CTL responses to focus immune responses on vaccine targets. To systematically investigate this hypothesis, we preimmunized mice with LCMV-WT, PICV-WT, CAND-WT, or WT Mopeia virus (MOPV-WT) and studied the animals' ability of responding to artLCMV-E7E6 vaccination 1 month later. MOPV is an Old World mammarenavirus and, thus, has a close genealogic relationship with LCMV, whereas the New World viruses PICV and CAND are only distantly related to LCMV (Figure 1D). Because of epitope sequence homology between LCMV and MOPV (Figure 5A), both viruses elicited clearly detectable CTL responses to NP396 whereas the more distantly related PICV-WT and CAND-WT did not (Figure 5B). Pre-existing immunity to LCMV or MOPV almost completely abrogated E7-specific CTL induction by artLCMV-E7E6, whereas the responses of PICV-WT- or CAND-WT-immune mice were only modestly below those of control mice without prior arenavirus infection (Figure 5C). This interference by LCMV-WT and MOPV-WT immunity was accompanied by high-frequency NP396-directed responses and a biased E7:NP396 immunodominance hierarchy upon artLCMV-E7E6 vaccination (Figures 5D and 5E). We relied on the immunodominant NP396 epitope as an indicator of backbone cross-reactivity, which likely comprised additional epitopes in the four viral backbone proteins NP, GP, L, and Z.

Next, we tested whether these interference and immunodominance hierarchies correlated with the immunogenicity of heterologous artARENA vector prime-boost combinations. To further expand the quiver of artARENA vectors, we generated a reverse genetic system for MOPV and, based thereupon, developed an artMOPV-P1A vector. Cell culture experiments demonstrated attenuated growth of artMOPV-P1A compared with its parental virus (Figure S7), analogous to the artARENA vectors described in Figures 1E–1G. We compared the ability of artLCMV-P1A to boost P1A-specific CTL responses induced by artPICV-P1A, artCAND-P1A, or artMOPV-P1A in BALB/c mice. Animals undergoing homologous artLCMV-P1A, artPICV-P1A, or artCAND-P1A prime-boost immunizations served as comparators and, after boost, remained in the 10% range or below (Figure 5F). Among the heterologous artARENA vector combinations, artPICV-P1A- and artCAND-P1A-primed animals were boosted efficiently by artLCMV-P1A, reaching frequencies in the 20%–30% range. Conversely, P1A-specific CTL responses of artMOPV-P1A-primed and artLCMV-P1A-boosted animals remained below 3%, failing to exceed the responses upon homologous artLCMV-P1A prime-boost. This indicated that the immunogenic benefit of heterologous prime-boost over homologous prime-boost was abolished when the closely related MOPV and LCMV vector backbones were combined. The above hierarchy of heterologous prime-boost combinations correlated inversely with the LCMV NP118-specific CTL responses to prime and boost (Figure 5G). artPICV-P1A and artCAND-P1A prime did not induce NP118-specific CTLs above technical background and repressed NP118-directed responses upon artLCMV-P1A boost. Conversely, artMOPV-P1A-primed and artLCMV-P1A-boosted animals had more than 50% NP118-specific $CD8^+$ T cells in peripheral blood with a corresponding P1A:NP118 epitope dominance ratio of less than 0.1 (Figure 5H). Thus, genealogic vector backbone relatedness and vector backbone-biased CTL responses correlated with inefficient induction of P1A-specific responses.

Interference by vector backbone-specific CTLs rather than by nAbs

Next, we investigated the induction and cross-reactive neutralizing activity of artARENA vector-induced antibody responses. Homologous prime-boost immunization with artCAND induced sizeable vector-nAb titers (Figure 6A). We failed to detect artLCMV- or artPICV-nAbs after homologous prime-boost immunization (Figure 6A), and neither artLCMV nor artPICV or artCAND immune sera cross-neutralized any of the other viruses. nAb induction by artCAND, but not artLCMV or artPICV, was in line with differentially dense glycan shields on the respective viruses' envelope proteins.[45]

These findings argued against vector-nAbs as a limiting factor in artLCMV- or artPICV-based homologous or heterologous prime-boost immunization. To formally rule out antibody-mediated inhibition, we performed homologous and heterologous prime-boost immunizations in B cell-deficient mice (JHT mice) and WT control mice. E7-specific responses to artPICV-E7E6 and artLCMV-E7E6 prime were indistinguishable in the two strains of mice (Figure 6B). More importantly, however, JHT and WT mice had a virtually identical response pattern to homologous artLCMV-E7E6 or heterologous artPICV-E7E6 boost, respectively. The superior immunogenicity of heterologous compared with homologous prime-boost despite a lack of anti-vector antibodies in JHT mice excluded anti-vector antibody responses as a major limitation in homologous artLCMV prime-boost immunization (Figure 6B).

To address whether anti-vector CTL responses, independently of other components of anti-vector immunity, can interfere with artLCMV-based vaccination, we "preimmunized" mice with a recombinant vaccinia virus expressing the NP396 epitope as a minigene (VACC-NP396). Control animals were either given vaccinia virus expressing an irrelevant transgene

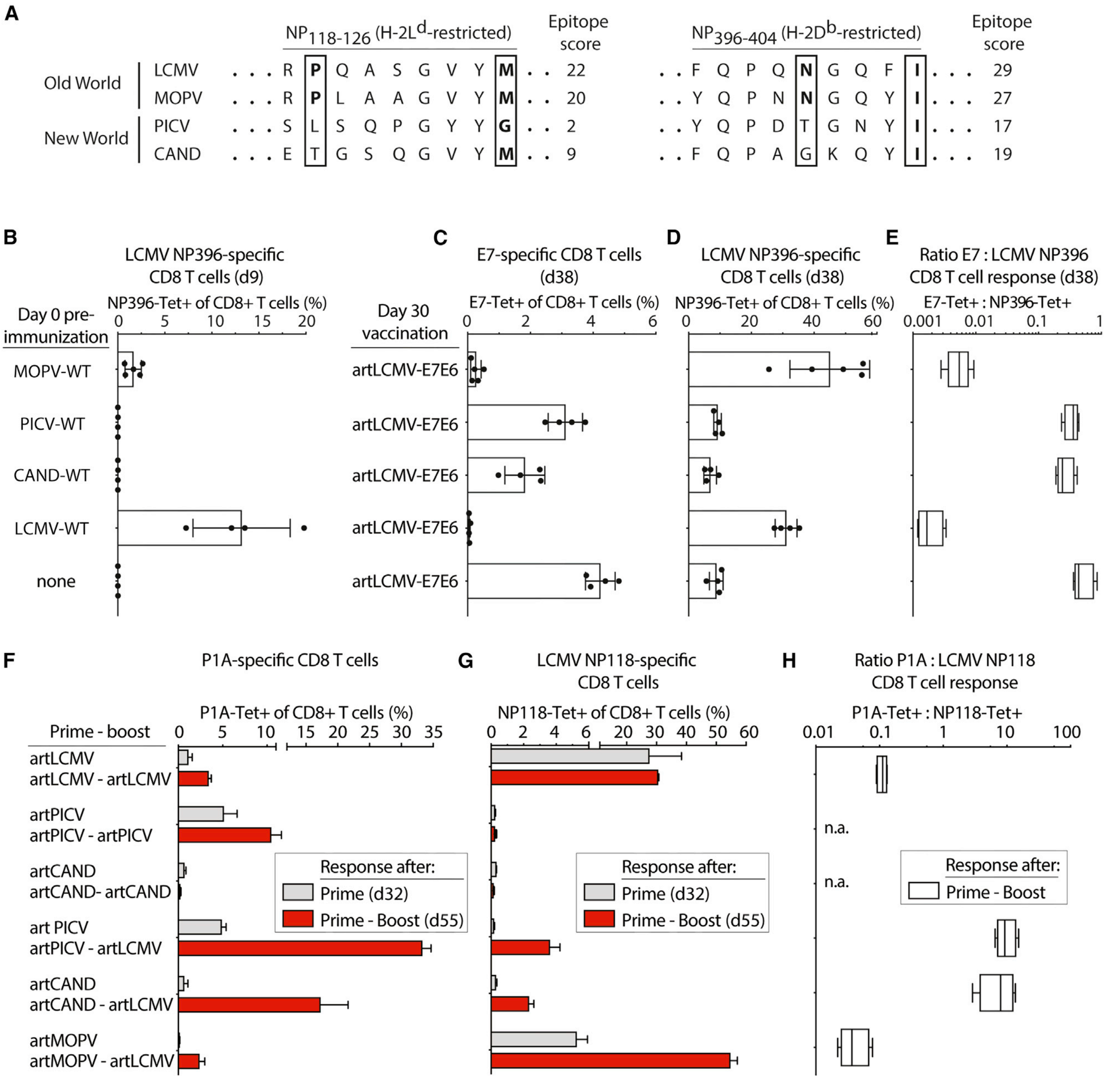

Figure 5. Genealogic artARENA vector backbone relatedness dictates interference by pre-existing immunity and potency in heterologous prime-boost immunization

(A) Alignment of the LCMV NP118-126 and NP396-404 epitope sequences with the respective homologous sequences in the Old World arenavirus MOPV and the New World arenaviruses PICV and CAND. Epitope scores were predicted by the SYFPEITHI algorithm.[70] Major histocompatibility complex (MHC) anchor positions are boxed, and amino acids corresponding to consensus anchor residues for H-2L^d and H-2D^b, respectively, are shown in bold.[71]

(B–E) We preimmunized C57BL/6 mice on day 0 with 10e5 PFUs of MOPV-WT, PICV-WT, CAND-WT, or LCMV-WT i.v. or left them uninfected. On day 9, we determined NP396-specific $CD8^+$ T cell frequencies in peripheral blood (B). On day 30, all mice were vaccinated with artLCMV-E7E6. Frequencies of E7-specific (C) and NP396-specific (D) $CD8^+$ T cells in the blood on day 38 served to calculate the epitope dominance ratio as displayed in (E). Symbols in (B)–(D) represent individual mice, and bars in (B)–(D) show their mean ± SD.

(F–H) We immunized BALB/c mice with 10e5 PFUs of artARENA-P1A vectors i.v. in various homologous and heterologous prime (day 0) to boost (day 35) combinations as indicated. On day 32 after prime and on day 55 (20 days after boost), we determined the frequencies of P1A-specific (F) and NP118-specific (G) $CD8^+$ T cells in the blood and calculated the P1A:NP118 epitope dominance ratio on day 55 (H). The lack of NP118-specific responses over technical background in artPICV-artPICV and artCAND-artCAND immunized mice precluded this latter assessment ("n.a."). Bars in (F) and (G) represent the mean ± SEM of 3 (artPICV-artPICV) to 4 mice (other groups). Boxes in (E) and (H) display the minimal and maximal values. N = 2.

See also Figure S7.

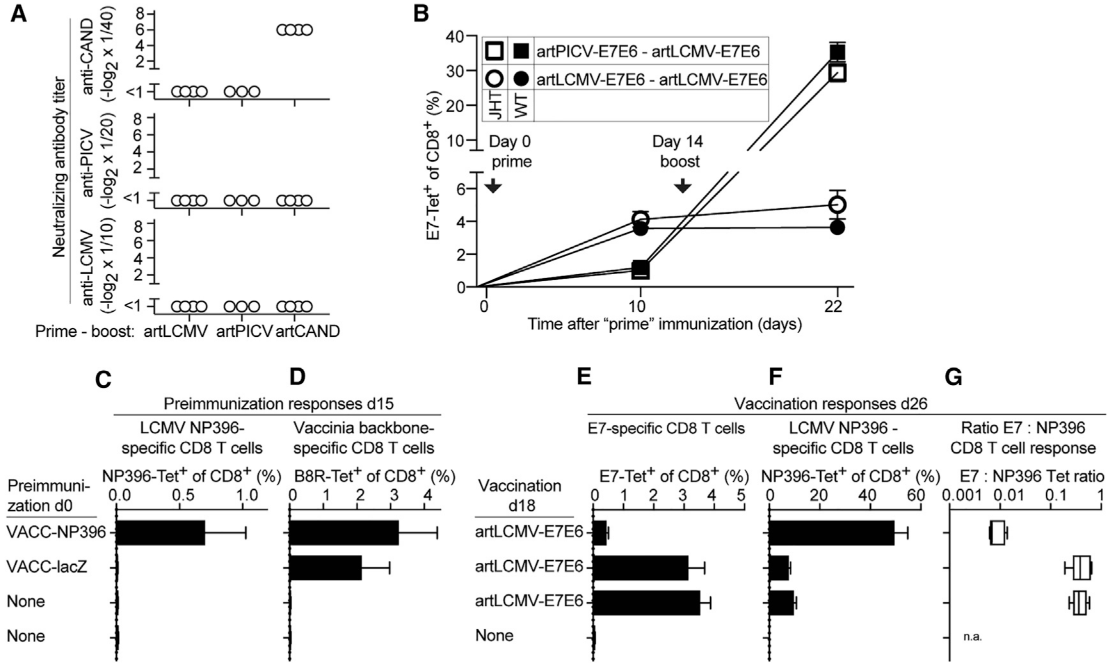

Figure 6. Interference by vector backbone-specific CTLs rather than by nAbs

(A) We immunized BALB/c mice i.v. with artLCMV-, artPICV-, or artCAND-based vectors on day 0 and day 35. Sera collected on day 45 (day 10 after homologous boost) were assayed for neutralizing activity against LCMV, PICV, and CAND. Symbols represent individual mice. One representative of two similar experiments is shown.

(B) We immunized B cell-deficient mice (JHT mice) and B cell-sufficient WT control mice with artLCMV-E7E6 and artPICV-E7E6 in homologous and heterologous prime-boost combinations on day 0 and day14 as indicated in the chart. The frequencies of E7-specific CD8$^+$ T cells in the blood was determined on day 10 (after prime) and day 22 (after boost). Symbols show the mean ± SEM of 4 mice. N = 2.

(C–G) On day 0, we preimmunized C57BL/6 mice i.v. with VACC-NP396 or VACC-lacZ or left them without preimmunization ("none"). On day 15, we determined LCMV NP396-specific (C) and vaccinia B8R-specific (D) CTLs in the blood by MHC class I tetramer staining. On day 18, i.v. artLCMV-E7E6 vaccination was performed. Eight days later (day 26), we determined E7-specific (E) as well as NP396-specific (F) CD8$^+$ T cell frequencies in the blood and calculated the E7:NP396 epitope dominance ratio (G). Bars represent the mean ± SEM of 5 mice per group. Boxes display the minimal and maximal values. N = 2.

(Vacc-lacZ) as preimmunization or were left unimmunized. As expected, VACC-NP396 preimmunization, but not VACC-lacZ, induced a CTL response to NP396 (Figure 6C), whereas both viruses triggered CTLs to the immunodominant vaccinia virus backbone epitope $B8R_{20-27}$ (Figure 6D). When subsequently vaccinated with artLCMV-E7E6, the E7-specific CTL responses of VACC-NP396-preimmune animals were 7- to 8-fold lower than those of VACC-lacZ-preimmune mice or controls not previously exposed to vaccinia virus (Figure 6E). Conversely, the NP396-specific CTL response of VACC-NP396-preimmune mice approached 50% of the circulating CD8$^+$ T cell pool, vastly exceeding the responses of VACC-lacZ-preimmune or vaccinia virus-naive mice (Figure 6F). Thus, pre-existing immunity to one immunodominant CD8 T cell epitope in the artLCMV backbone was sufficient to repress E7-directed CTL responses upon vaccination with a concomitant shift in E7:NP396 epitope dominance (Figure 6G).

Heterologous artARENA vector immunotherapy increases TIL numbers and tumor cure rates, resulting in long-term anti-tumor immunity

To assess whether the augmented immunogenicity of heterologous artARENA vector prime-boost translated into superior therapeutic efficacy, we exploited two transplantable syngeneic mouse tumor models. The P815 mastocytoma cell line is derived from a DBA/2 mouse and expresses the P1A cancer-testis antigen, whereas the C57BL/6-derived TC-1 cell lines serves as a model of HPV16 E7E6-expressing cancer. We implanted P815 and TC-1 tumors subcutaneously into the flank of mice and initiated artPICV or artLCMV vector therapy when tumors were palpable (P815, day 9) or had reached an average critical volume (~100 mm^3; TC-1, day 8). artPICV-artLCMV heterologous therapy delivering the respective tumor antigen, P1A or E7E6, was compared with homologous artPICV-artPICV or artLCMV-artLCMV prime-boost, all administered at an interval of 7 days (P815 model) or 10 days (TC-1 model). Homologous artPICV and homologous artLCMV immunotherapy afforded clear tumor volume control compared with untreated control animals (Figures 7A and 7B). In both tumor models, however, the most pronounced and durable effect on tumor volume was seen upon heterologous artPICV-artLCMV therapy. This therapeutic effect depended on the vectorized antigen; a control group of P815 tumor-bearing mice that were given artPICV-GFP-artLCMV-GFP prime-boost, delivering the irrelevant GFP transgene instead of a TAA, did not show a clear therapeutic effect. Homologous prime-boost with TAA-expressing artLCMV or artPICV extended

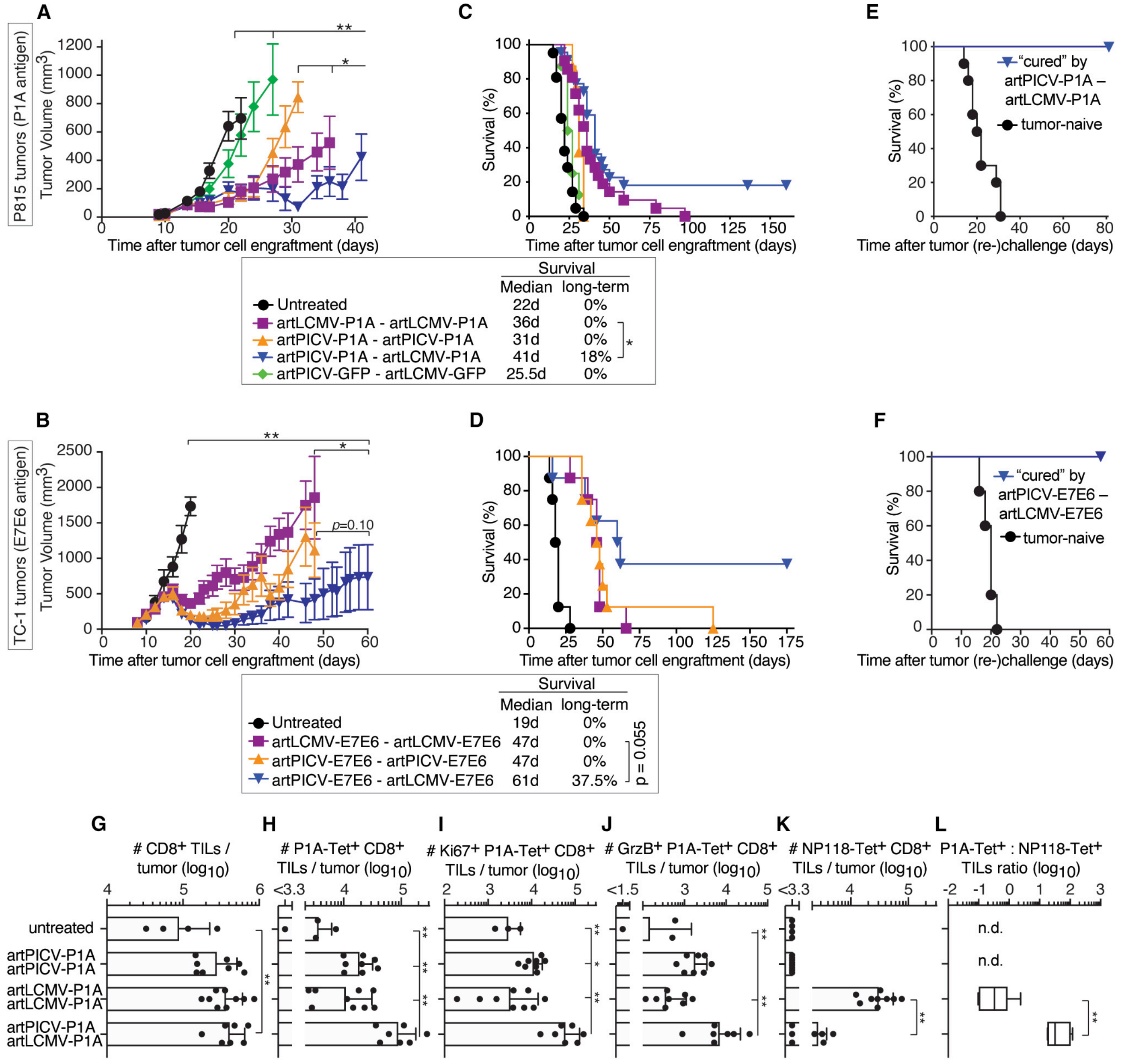

Figure 7. Heterologous artARENA vector immunotherapy increases TIL numbers and tumor cure rates, resulting in long-term anti-tumor immunity

(A–L) We implanted tumor cells subcutaneously in the flanks of mice. (A), (C), (E), and (G–L): DBA/2 mice, P815 tumor cells. (B), (D), and (F): C57BL/6 mice, TC-1 tumor cells. When animals exhibited palpable tumor masses on day 9 (P815) or when tumors had reached an average volume of ~100 mm^3 on day 8 (TC-1), mice were assigned randomly to the indicated prime-boost regimens administered i.v. on day 9 and day 16 (P815) or day 8 and day 18 (TC-1).

(A and B) Symbols show tumor volumes (mean ± SEM) from two (A) and one (B) independent experiments. The curves end when more than 50% of animals in a group have reached humane endpoints. Entire tumor volume curves were statistically compared as described in STAR methods.

(C and D) Kaplan-Meier survival curves of the animals, with median survival and percent long-term survivors indicated.

(A and C) n = 21 (untreated), 21 (artLCMV-P1A-artLCMV-P1A), 8 (artPICV-P1A-artPICV-P1A), 22 (artPICV-P1A-artLCMV-P1A), and 8 (artPICV-GFP-artLCMV-GFP).

(B and D) n = 8. *$p < 0.05$ by two-tailed chi-square test.

(E and F) Animals, which had rejected their tumors ("cured") and tumor-naive controls were re-challenged subcutaneously with tumor cells on day 160 and day 140 (E) or day 118 (F) after primary tumor implantation. Re-challenged mice did not form palpable tumors (data not shown).

(E) Combined data from tumor-free mice (n = 5) and tumor-naive mice (n = 10) in two independent experiments.

(F) Tumor-free mice (n = 3) and tumor-naive mice (n = 5) from one experiment.

(legend continued on next page)

CellPress
OPEN ACCESS

the survival of tumor-bearing mice, albeit to a lesser extent than heterologous prime-boost, and all mice receiving homologous prime-boost immunization eventually reached humane endpoints (Figures 7C and 7D). In contrast, 18 percent of mice with P815 tumors and 37.5 percent of animals with TC-1 tumors rejected their respective tumors when undergoing heterologous TAA-vectorizing artPICV-artLCMV immunotherapy (P1A and E7E6 but not GFP), resulting in the animals' survival for more than 125 days (Figures 7C and 7D). When these long-term survivors were re-challenged with the same tumor cells, no tumor growth was recorded, whereas tumor- and therapy-naive control animals progressed rapidly to humane endpoints (Figures 7E and 7F). This observation indicated that elimination of established tumors by artPICV-artLCMV immunotherapy resulted in long-term anti-tumor immunity.

We enumerated and characterized tumor-infiltrating CTLs on day 5 after homologous or heterologous artARENA vector immunotherapy of P815 tumors (Figures 7G–7L). Heterologous artPICV-P1A-artLCMV-P1A immunotherapy did not substantially augment total tumor-infiltrating CTL numbers compared with homologous immunotherapy with either one of these vectors (Figure 7G), but P1A-specific CTLs in tumors were significantly more numerous upon heterologous compared with homologous artARENA immunotherapy (Figure 7H). In line with this observation, we found an increase in proliferating (Ki67$^+$) and granzyme B-expressing (cytotoxic) P1A-specific, tumor-infiltrating CTLs in mice with heterologous artARENA immunotherapy (Figures 7I and 7J). Heterologous artPICV-P1A-artLCMV-P1A therapy triggered significantly less NP118-specific TILs than homologous artLCMV-P1A prime-boost vaccination (Figure 7K), with a corresponding shift in P1A:NP118 epitope dominance in the tumor (Figure 7L). These data indicated that heterologous artARENA vector immunization shifted the TAA:vector backbone immunodominance patterns of tumor-infiltrating CTLs.

DISCUSSION

Elicitation of clinically effective tumor-specific CTL responses remains an important unmet medical need. Here we identify epitope dominance and interference by vector backbone-directed T cell responses as an important hurdle in vectored vaccine delivery. Based on this concept in conjunction with arenaviral genealogy analyses, we developed potent heterologous artARENA-based immunization regimens inducing effective anti-tumor immunity.

Therapeutic cancer vaccination is a particularly demanding field of active immunization. Induction of high-frequency CTL responses against TAAs is often encumbered by central as well as peripheral tolerance mechanisms, limiting the available T cell repertoire and its responsiveness.[72] Additional challenges arise from chronic antigenic exposure, which can lead to functional adaptation or impairment of specific T cell responses.[73–75] In this context, potent viral vector systems and their optimal combination are of particular importance. Although poxvirus delivery systems have been evaluated clinically as cancer vaccines for more than 2 decades,[18,76] a wide variety of viral vector systems has entered clinical testing in recent years. These approaches comprise alphavirus vectors,[20] human and sAd-based vectors,[21,22] lentivirus delivery systems,[23] rhabdovirus vectors,[24,25] as well as combinations thereof, attesting to the promise of and broad interest in virally vectored cancer vaccination.

Animal studies have shown that arenavirus vectors are immunogenic when administered intravenously, intradermally, subcutaneously, or intramuscularly,[41,43] and the latter route has also been validated clinically in a human trial.[44] Earlier studies from our lab revealed, however, that the IL-33-ST2 alarmin pathway contributes essentially to the effectiveness of artARENA-vectored cancer immunotherapy,[29] and the vector's ability to trigger this pathway correlates with its spread into IL-33-expressing splenic stromal cells. In line with other investigators' work,[77] these findings highlight the importance of vaccine delivery to specialized compartments of secondary lymphoid organs. The intravenous route provides optimal access to these tissues and, therefore, has been used here; it is also exploited in an ongoing clinical trial for artLCMV-based therapy of HPV16-positive head and neck cancer.[49]

A discriminating feature of artLCMV and artPICV vector technology is the lack of vector-nAb induction[29,41,43,44] because of the viral glycan shield[45] and globally low arenavirus seroprevalence.[78–81] The reason for the latter is the natural host range of mammarenaviruses being restricted to rodents, combined with only rare transmission from rodents to humans. Although they do not elicit nAbs against their own glycan-shielded envelope protein, replication-deficient rLCMV vectors elicit potent antibody immunity against vectorized cargo.[29,41,43–45] While future work should investigate the utility of artARENA vector technology for prophylactic antibody induction against infectious diseases, potent CD8 T cell responses position heterologous artARENA vector prime-boost immunization as a promising strategy for therapeutic vaccination in persistent microbial diseases, notably HIV and hepatitis B.[7,82]

Our findings highlight anti-vector T cell immunity as a mechanism of interference in artARENA vector homologous prime-boost vaccination. For delivery systems such as Ad and poxvirus vectors, which readily elicit potent vector-nAbs,[32,83] the latter are commonly taken as surrogate and supposed main mechanisms of interference by anti-vector immunity. Several observations suggest, however, that pre-existing anti-vector T cells impede responses to Ad and poxvirus vector systems, too. Inefficient responses to MVA-vectored TAAs has been accredited to epitope dominance and competition by vector backbone-directed T cell responses.[84] Similarly, the inhibitory activity of Ad vector-nAbs[83] may have obscured the contribution of additional interference mechanisms. Certain pairs of adenoviral vectors, although serologically distinct, failed to effectively boost each other.[36,37] This indicates that the mere absence of vector-nAbs cannot predict efficient boosting. Conversely, pre-existing Ad-specific T cell immunity is correlated inversely with

(G–L) We analyzed TILs in P815 tumors on day 20 (day 4 after artARENA-P1A vector boost). Total CD8$^+$ TILs (G); P1A-specific CD8$^+$ TILs (H), among them Ki67$^+$ (I) or granzyme B-expressing cells (J); and NP118-specific TILs (K) were enumerated. The P1A: NP118 epitope dominance ratio was calculated (L). Symbols represent individual mice, bars show the mean, and error bars indicate SEM. Boxes display the minimal and maximal values.

HIV-specific CTL induction by rAd5 vectors in human vaccine trials.[85] Pre-existing T cell reactivity to Ad5 is found in more than 80% of healthy adults, irrespective of Ad5-specific serostatus.[86] It originates from prior exposure to Ads of unrelated serotypes, targets epitopes in conserved regions of the viral genome,[87] and expands significantly upon rAd5-vectored vaccination.[86] These considerations highlight advantages of viral vector platforms based on virus families such as Arenaviridae, which circulate almost exclusively in the animal kingdom.

Limitations of study

We relied on the transgene:backbone epitope dominance ratio of CD8 T cell responses as an indicator and correlate of backbone-directed T cell interference. Besides inter-clonal competition of transgene- and backbone-specific T cells,[88] an alternative and not mutually exclusive mechanism may consist of accelerated elimination of vector-transduced APCs by pre-existing anti-vector T cells.[89]

The present data obtained for mice may imperfectly predict efficacy in humans, but they call for immediate clinical evaluation of heterologous artPICV-artLCMV prime-boost immunization regimens to benefit patients. artPICV-E7E6 and artLCMV-E7E6 are currently entering clinical phase 1 testing as a repeated alternating prime-boost immunization regimen for HPV16-positive head and neck cancer.[49] Irrespective of the limited predictive value of transplantable mouse tumor models, our findings regarding potent CTL induction to self-antigens such as P1A suggest that self-tolerance can be broken efficiently and provide an incentive for our plan of applying artARENA vector technology to a broader range of cancers, most of which do not express viral target antigens. The arenavirus vector platform accommodates transgenes up to ~2,000 bp,[29,43] lending itself for delivery of all types of proteinaceous cancer targets,[90] but it remains unknown which ones provide the best clinical efficacy.

STAR★METHODS

Detailed methods are provided in the online version of this paper and include the following:

- KEY RESOURCES TABLE
- RESOURCE AVAILABILITY
  - Lead contact
  - Materials availability
  - Data and code availability
- EXPERIMENTAL MODEL AND SUBJECT DETAILS
  - Animals and ethics statement
  - Cell lines
- METHOD DETAILS
  - Viruses, titration and neutralization test
  - Viral virulence testing
  - Tumor implantation and tumor measurement
  - Virus engineering, infection and immunization
  - Assessment of blood-brain-barrier integrity
  - Virus sequencing and genealogy tree building
  - Flow cytometry
- QUANTIFICATION AND STATISTICAL ANALYSIS
  - Statistical testing

SUPPLEMENTAL INFORMATION

Supplemental Information can be found online at https://doi.org/10.1016/j.xcrm.2021.100209.

ACKNOWLEDGMENTS

We wish to thank Nadège Lagarde, Beatrice Dolder Schlienger, Felix Stemeseder, and Sophie Schulha for excellent technical assistance and Theresa Kleissner, Goran Bekic, Daniel Oeler, and Sonja Feher for diligent implementation of animal studies. This work was supported by the Swiss National Science Foundation (310030_185318/1 to D.D.P., CRSII3_160772/1 to D.D.P. and D.M., 310030_173010 to D.M., and CRSII5_170929 to A.Z.), Hookipa Pharma, Inc. (to D.D.P., A.Z., and D.M.), and by Austrian Research Promotion Agency (Österreichische Forschungsförderungsgesellschaft [FFG]).

AUTHOR CONTRIBUTIONS

W.V.B., N.K., A.-F.M., S.M.K., S.S., J.R., U.B., I.V., S.G., C.M., D.M., K.K.O., A.Z., and D.D.P. contributed to experimental conception and design. W.V.B., N.K., A.-F.M., S.M.K., M.A.K., M.L., S.D., J.R., I.V., M.P., R.K., S.H., and C.M. performed experiments. W.V.B., N.K., A.-F.M., S.M.K., S.S., J.R., U.B., I.V., M.P., C.M., D.M., K.K.O., A.Z., and D.D.P. analyzed and/or interpreted the data. W.V.B., N.K., A.Z., and D.D.P. wrote the manuscript.

DECLARATION OF INTERESTS

S.S., J.R., U.B., and K.K.O. are employees and stock option holders of Hookipa Pharma, Inc. D.D.P. is a founder, consultant, shareholder, and stock option holder of Hookipa Pharma, Inc. W.V.B., S.M.K., S.S., S.D., U.B., D.D.P., D.M., and K.K.O. are listed as inventors on patents describing artARENA vector technology. W.V.B. and D.D.P. are married.

Received: September 15, 2020
Revised: December 16, 2020
Accepted: February 4, 2021
Published: March 3, 2021

REFERENCES

1. Zhang, L., Conejo-Garcia, J.R., Katsaros, D., Gimotty, P.A., Massobrio, M., Regnani, G., Makrigiannakis, A., Gray, H., Schlienger, K., Liebman, M.N., et al. (2003). Intratumoral T cells, recurrence, and survival in epithelial ovarian cancer. N. Engl. J. Med. *348*, 203–213.
2. Pagès, F., Berger, A., Camus, M., Sanchez-Cabo, F., Costes, A., Molidor, R., Mlecnik, B., Kirilovsky, A., Nilsson, M., Damotte, D., et al. (2005). Effector memory T cells, early metastasis, and survival in colorectal cancer. N. Engl. J. Med. *353*, 2654–2666.
3. Galon, J., Costes, A., Sanchez-Cabo, F., Kirilovsky, A., Mlecnik, B., Lagorce-Pagès, C., Tosolini, M., Camus, M., Berger, A., Wind, P., et al. (2006). Type, density, and location of immune cells within human colorectal tumors predict clinical outcome. Science *313*, 1960–1964.
4. Tumeh, P.C., Harview, C.L., Yearley, J.H., Shintaku, I.P., Taylor, E.J., Robert, L., Chmielowski, B., Spasic, M., Henry, G., Ciobanu, V., et al. (2014). PD-1 blockade induces responses by inhibiting adaptive immune resistance. Nature *515*, 568–571.
5. Jin, X., Bauer, D.E., Tuttleton, S.E., Lewin, S., Gettie, A., Blanchard, J., Irwin, C.E., Safrit, J.T., Mittler, J., Weinberger, L., et al. (1999). Dramatic rise in plasma viremia after CD8(+) T cell depletion in simian immunodeficiency virus-infected macaques. J. Exp. Med. *189*, 991–998.
6. Schmitz, J.E., Kuroda, M.J., Santra, S., Sasseville, V.G., Simon, M.A., Lifton, M.A., Racz, P., Tenner-Racz, K., Dalesandro, M., Scallon, B.J., et al. (1999). Control of viremia in simian immunodeficiency virus infection by CD8+ lymphocytes. Science *283*, 857–860.

CellPress
OPEN ACCESS

7. Kosinska, A.D., Bauer, T., and Protzer, U. (2017). Therapeutic vaccination for chronic hepatitis B. Curr. Opin. Virol. *23*, 75–81.

8. Romero, P., Banchereau, J., Bhardwaj, N., Cockett, M., Disis, M.L., Dranoff, G., Gilboa, E., Hammond, S.A., Hershberg, R., Korman, A.J., et al. (2016). The Human Vaccines Project: A roadmap for cancer vaccine development. Sci. Transl. Med. *8*, 334ps339.

9. Melief, C.J.M. (2017). Cancer: Precision T-cell therapy targets tumours. Nature *547*, 165–167.

10. Kantoff, P.W., Higano, C.S., Shore, N.D., Berger, E.R., Small, E.J., Penson, D.F., Redfern, C.H., Ferrari, A.C., Dreicer, R., Sims, R.B., et al.; IMPACT Study Investigators (2010). Sipuleucel-T immunotherapy for castration-resistant prostate cancer. N. Engl. J. Med. *363*, 411–422.

11. Vansteenkiste, J.F., Cho, B.C., Vanakesa, T., De Pas, T., Zielinski, M., Kim, M.S., Jassem, J., Yoshimura, M., Dahabreh, J., Nakayama, H., et al. (2016). Efficacy of the MAGE-A3 cancer immunotherapeutic as adjuvant therapy in patients with resected MAGE-A3-positive non-small-cell lung cancer (MAGRIT): a randomised, double-blind, placebo-controlled, phase 3 trial. Lancet Oncol. *17*, 822–835.

12. Butts, C., Socinski, M.A., Mitchell, P.L., Thatcher, N., Havel, L., Krzakowski, M., Nawrocki, S., Ciuleanu, T.E., Bosquée, L., Trigo, J.M., et al.; START trial team (2014). Tecemotide (L-BLP25) versus placebo after chemoradiotherapy for stage III non-small-cell lung cancer (START): a randomised, double-blind, phase 3 trial. Lancet Oncol. *15*, 59–68.

13. Gulley, J.L., Borre, M., Vogelzang, N.J., Ng, S., Agarwal, N., Parker, C.C., Pook, D.W., Rathenborg, P., Flaig, T.W., Carles, J., et al. (2019). Phase III Trial of PROSTVAC in Asymptomatic or Minimally Symptomatic Metastatic Castration-Resistant Prostate Cancer. J. Clin. Oncol. *37*, 1051–1061.

14. Rosenberg, S.A., Yang, J.C., and Restifo, N.P. (2004). Cancer immunotherapy: moving beyond current vaccines. Nat. Med. *10*, 909–915.

15. Gajewski, T.F., Schreiber, H., and Fu, Y.X. (2013). Innate and adaptive immune cells in the tumor microenvironment. Nat. Immunol. *14*, 1014–1022.

16. Joyce, J.A., and Fearon, D.T. (2015). T cell exclusion, immune privilege, and the tumor microenvironment. Science *348*, 74–80.

17. Zamarin, D., Holmgaard, R.B., Subudhi, S.K., Park, J.S., Mansour, M., Palese, P., Merghoub, T., Wolchok, J.D., and Allison, J.P. (2014). Localized oncolytic virotherapy overcomes systemic tumor resistance to immune checkpoint blockade immunotherapy. Sci. Transl. Med. *6*, 226ra32.

18. Tsang, K.Y., Zaremba, S., Nieroda, C.A., Zhu, M.Z., Hamilton, J.M., and Schlom, J. (1995). Generation of human cytotoxic T cells specific for human carcinoembryonic antigen epitopes from patients immunized with recombinant vaccinia-CEA vaccine. J. Natl. Cancer Inst. *87*, 982–990.

19. Kantoff, P.W., Schuetz, T.J., Blumenstein, B.A., Glode, L.M., Bilhartz, D.L., Wyand, M., Manson, K., Panicali, D.L., Laus, R., Schlom, J., et al. (2010). Overall survival analysis of a phase II randomized controlled trial of a Poxviral-based PSA-targeted immunotherapy in metastatic castration-resistant prostate cancer. J. Clin. Oncol. *28*, 1099–1105.

20. Morse, M.A., Hobeika, A.C., Osada, T., Berglund, P., Hubby, B., Negri, S., Niedzwiecki, D., Devi, G.R., Burnett, B.K., Clay, T.M., et al. (2010). An alphavirus vector overcomes the presence of neutralizing antibodies and elevated numbers of Tregs to induce immune responses in humans with advanced cancer. J. Clin. Invest. *120*, 3234–3241.

21. Aurisicchio, L., Fridman, A., Mauro, D., Sheloditna, R., Chiappori, A., Bagchi, A., and Ciliberto, G. (2020). Safety, tolerability and immunogenicity of V934/V935 hTERT vaccination in cancer patients with selected solid tumors: a phase I study. J. Transl. Med. *18*, 39.

22. clinicaltrials.gov (2020). A Phase 1 Study To Evaluate Escalating Doses Of A Vaccine-Based Immunotherapy Regimen For Prostate Cancer (PrCa VBIR). https://clinicaltrialsgov/ct2/show/NCT02616185.

23. Pollack, S.M., Lu, H., Gnjatic, S., Somaiah, N., O'Malley, R.B., Jones, R.L., Hsu, F.J., and Ter Meulen, J. (2017). First-in-Human Treatment With a Dendritic Cell-targeting Lentiviral Vector-expressing NY-ESO-1, LV305, Induces Deep, Durable Response in Refractory Metastatic Synovial Sarcoma Patient. J. Immunother. *40*, 302–306.

24. Atherton, M.J., Stephenson, K.B., Nikota, J.K., Hu, Q.N., Nguyen, A., Wan, Y., and Lichty, B.D. (2018). Preclinical development of peptide vaccination combined with oncolytic MG1-E6E7 for HPV-associated cancer. Vaccine *36*, 2181–2192.

25. clinicaltrials.gov (2020). This is a Trial of MG1-E6E7 With Ad-E6E7 and Atezolizumab in Patients With HPV Associated Cancers (Kingfisher). https://clinicaltrials.gov/ct2/show/NCT03618953.

26. Pinschewer, D.D. (2017). Virally vectored vaccine delivery: medical needs, mechanisms, advantages and challenges. Swiss Med. Wkly. *147*, w14465.

27. Querec, T., Bennouna, S., Alkan, S., Laouar, Y., Gorden, K., Flavell, R., Akira, S., Ahmed, R., and Pulendran, B. (2006). Yellow fever vaccine YF-17D activates multiple dendritic cell subsets via TLR2, 7, 8, and 9 to stimulate polyvalent immunity. J. Exp. Med. *203*, 413–424.

28. Peine, M., Marek, R.M., and Löhning, M. (2016). IL-33 in T Cell Differentiation, Function, and Immune Homeostasis. Trends Immunol. *37*, 321–333.

29. Kallert, S.M., Darbre, S., Bonilla, W.V., Kreutzfeldt, M., Page, N., Müller, P., Kreuzaler, M., Lu, M., Favre, S., Kreppel, F., et al. (2017). Replicating viral vector platform exploits alarmin signals for potent CD8(+) T cell-mediated tumour immunotherapy. Nat. Commun. *8*, 15327.

30. Baumann, C., Bonilla, W.V., Fröhlich, A., Helmstetter, C., Peine, M., Hegazy, A.N., Pinschewer, D.D., and Löhning, M. (2015). T-bet- and STAT4-dependent IL-33 receptor expression directly promotes antiviral Th1 cell responses. Proc. Natl. Acad. Sci. USA *112*, 4056–4061.

31. Bonilla, W.V., Fröhlich, A., Senn, K., Kallert, S., Fernandez, M., Johnson, S., Kreutzfeldt, M., Hegazy, A.N., Schrick, C., Fallon, P.G., et al. (2012). The alarmin interleukin-33 drives protective antiviral $CD8^+$ T cell responses. Science *335*, 984–989.

32. Pittman, P.R., Hahn, M., Lee, H.S., Koca, C., Samy, N., Schmidt, D., Hornung, J., Weidenthaler, H., Heery, C.R., Meyer, T.P.H., et al. (2019). Phase 3 Efficacy Trial of Modified Vaccinia Ankara as a Vaccine against Smallpox. N. Engl. J. Med. *381*, 1897–1908.

33. Kündig, T.M., Kalberer, C.P., Hengartner, H., and Zinkernagel, R.M. (1993). Vaccination with two different vaccinia recombinant viruses: long-term inhibition of secondary vaccination. Vaccine *11*, 1154–1158.

34. McElrath, M.J., De Rosa, S.C., Moodie, Z., Dubey, S., Kierstead, L., Janes, H., Defawe, O.D., Carter, D.K., Hural, J., Akondy, R., et al.; Step Study Protocol Team (2008). HIV-1 vaccine-induced immunity in the test-of-concept Step Study: a case-cohort analysis. Lancet *372*, 1894–1905.

35. Colloca, S., Barnes, E., Folgori, A., Ammendola, V., Capone, S., Cirillo, A., Siani, L., Naddeo, M., Grazioli, F., Esposito, M.L., et al. (2012). Vaccine vectors derived from a large collection of simian adenoviruses induce potent cellular immunity across multiple species. Sci. Transl. Med. *4*, 115ra112.

36. Barnes, E., Folgori, A., Capone, S., Swadling, L., Aston, S., Kurioka, A., Meyer, J., Huddart, R., Smith, K., Townsend, R., et al. (2012). Novel adenovirus-based vaccines induce broad and sustained T cell responses to HCV in man. Sci. Transl. Med. *4*, 115ra111.

37. Stanley, D.A., Honko, A.N., Asiedu, C., Trefry, J.C., Lau-Kilby, A.W., Johnson, J.C., Hensley, L., Ammendola, V., Abbate, A., Grazioli, F., et al. (2014). Chimpanzee adenovirus vaccine generates acute and durable protective immunity against ebolavirus challenge. Nat. Med. *20*, 1126–1129.

38. Zinkernagel, R.M. (2002). Lymphocytic choriomeningitis virus and immunology. Curr. Top. Microbiol. Immunol. *263*, 1–5.

39. Flatz, L., Bergthaler, A., de la Torre, J.C., and Pinschewer, D.D. (2006). Recovery of an arenavirus entirely from RNA polymerase I/II-driven cDNA. Proc. Natl. Acad. Sci. USA *103*, 4663–4668.

40. Sánchez, A.B., and de la Torre, J.C. (2006). Rescue of the prototypic Arenavirus LCMV entirely from plasmid. Virology *350*, 370–380.

41. Flatz, L., Hegazy, A.N., Bergthaler, A., Verschoor, A., Claus, C., Fernandez, M., Gattinoni, L., Johnson, S., Kreppel, F., Kochanek, S., et al. (2010). Development of replication-defective lymphocytic choriomeningitis virus vectors for the induction of potent CD8+ T cell immunity. Nat. Med. *16*, 339–345.

42. Flatz, L., Cheng, C., Wang, L., Foulds, K.E., Ko, S.Y., Kong, W.P., Roychoudhuri, R., Shi, W., Bao, S., Todd, J.P., et al. (2012). Gene-based vaccination with a mismatched envelope protects against simian immunodeficiency virus infection in nonhuman primates. J. Virol. *86*, 7760–7770.

43. Penaloza MacMaster, P., Shields, J.L., Alayo, Q.A., Cabral, C., Jimenez, J., Mondesir, J., Chandrashekar, A., Cabral, J.M., Lim, M., Iampietro, M.J., et al. (2017). Development of novel replication-defective lymphocytic choriomeningitis virus vectors expressing SIV antigens. Vaccine *35*, 1–9.

44. Schwendinger, M., Thiry, G., De Vos, B., Leroux-Roels, G., Bruhwyler, J., Huygens, A., Ganeff, C., Buchinger, H., Orlinger, K.K., Pinschewer, D.D., et al. (2020). A Randomized Dose-Escalating Phase I Trial of a Replication-Deficient Lymphocytic Choriomeningitis Virus Vector-Based Vaccine Against Human Cytomegalovirus. J. Infect. Dis. *2020*, jiaa121.

45. Sommerstein, R., Flatz, L., Remy, M.M., Malinge, P., Magistrelli, G., Fischer, N., Sahin, M., Bergthaler, A., Igonet, S., Ter Meulen, J., et al. (2015). Arenavirus Glycan Shield Promotes Neutralizing Antibody Evasion and Protracted Infection. PLoS Pathog. *11*, e1005276.

46. ClinicalTrials.gov (2021). A Study of CMV Vaccine (HB-101) in Kidney Transplant Patients. https://clinicaltrials.gov/ct2/show/NCT03629080.

47. Emonet, S.F., Garidou, L., McGavern, D.B., and de la Torre, J.C. (2009). Generation of recombinant lymphocytic choriomeningitis viruses with trisegmented genomes stably expressing two additional genes of interest. Proc. Natl. Acad. Sci. USA *106*, 3473–3478.

48. Dhanwani, R., Zhou, Y., Huang, Q., Verma, V., Dileepan, M., Ly, H., and Liang, Y. (2015). A Novel Live Pichinde Virus-Based Vaccine Vector Induces Enhanced Humoral and Cellular Immunity after a Booster Dose. J. Virol. *90*, 2551–2560.

49. ClinicalTrials.gov (2020). A Study of TheraT® Vector(s) Expressing HPV 16+ in Patients With HPV 16+ Confirmed Cancers (ClinicalTrials.gov). https://clinicaltrials.gov/ct2/show/NCT04180215.

50. Maiztegui, J.I., McKee, K.T., Jr., Barrera Oro, J.G., Harrison, L.H., Gibbs, P.H., Feuillade, M.R., Enria, D.A., Briggiler, A.M., Levis, S.C., Ambrosio, A.M., et al.; AHF Study Group (1998). Protective efficacy of a live attenuated vaccine against Argentine hemorrhagic fever. J. Infect. Dis. *177*, 277–283.

51. Buchmeier, M., Adam, E., and Rawls, W.E. (1974). Serological evidence of infection by Pichinde virus among laboratory workers. Infect. Immun. *9*, 821–823.

52. Armstrong, C. (1942). Some recent research in the field of neurotropic viruses with especial reference to lymphocytic choriomeningitis and herpes simplex. Mil. Surg. *91*, 129–146.

53. Stone, G.S., Glover, M., Jilg, N., and Sfeir, M.M. (2019). Case 40-2019: A 26-Year-Old Returning Traveler with Headache. N. Engl. J. Med. *381*, 2553–2560.

54. Souders, H.T., Byler, D., Marupudi, N., Patel, R., and McSherry, G. (2015). Protracted symptoms in lymphocytic choriomeningitis: a case report. J. Child Neurol. *30*, 644–647.

55. Cassetti, M.C., McElhiney, S.P., Shahabi, V., Pullen, J.K., Le Poole, I.C., Eiben, G.L., Smith, L.R., and Kast, W.M. (2004). Antitumor efficacy of Venezuelan equine encephalitis virus replicon particles encoding mutated HPV16 E6 and E7 genes. Vaccine *22*, 520–527.

56. Bergthaler, A., Gerber, N.U., Merkler, D., Horvath, E., de la Torre, J.C., and Pinschewer, D.D. (2006). Envelope exchange for the generation of live-attenuated arenavirus vaccines. PLoS Pathog. *2*, e51.

57. Pinschewer, D.D., Schedensack, M., Bergthaler, A., Horvath, E., Brück, W., Löhning, M., and Merkler, D. (2010). T cells can mediate viral clearance from ependyma but not from brain parenchyma in a major histocompatibility class I- and perforin-independent manner. Brain *133*, 1054–1066.

58. Christensen, J.E., de Lemos, C., Moos, T., Christensen, J.P., and Thomsen, A.R. (2006). CXCL10 is the key ligand for CXCR3 on CD8+ effector T cells involved in immune surveillance of the lymphocytic choriomeningitis virus-infected central nervous system. J. Immunol. *176*, 4235–4243.

59. Cole, G.A., Nathanson, N., and Prendergast, R.A. (1972). Requirement for theta-bearing cells in lymphocytic choriomeningitis virus-induced central nervous system disease. Nature *238*, 335–337.

60. Derbinski, J., Schulte, A., Kyewski, B., and Klein, L. (2001). Promiscuous gene expression in medullary thymic epithelial cells mirrors the peripheral self. Nat. Immunol. *2*, 1032–1039.

61. Uyttenhove, C., Godfraind, C., Lethé, B., Amar-Costesec, A., Renauld, J.C., Gajewski, T.F., Duffour, M.T., Warnier, G., Boon, T., and Van den Eynde, B.J. (1997). The expression of mouse gene P1A in testis does not prevent safe induction of cytolytic T cells against a P1A-encoded tumor antigen. Int. J. Cancer *70*, 349–356.

62. Huijbers, I.J., Soudja, S.M., Uyttenhove, C., Buferne, M., Inderberg-Suso, E.M., Colau, D., Pilotte, L., Powis de Tenbossche, C.G., Chomez, P., Brasseur, F., et al. (2012). Minimal tolerance to a tumor antigen encoded by a cancer-germline gene. J. Immunol. *188*, 111–121.

63. Böttcher, J.P., Beyer, M., Meissner, F., Abdullah, Z., Sander, J., Höchst, B., Eickhoff, S., Rieckmann, J.C., Russo, C., Bauer, T., et al. (2015). Functional classification of memory CD8(+) T cells by CX3CR1 expression. Nat. Commun. *6*, 8306.

64. Gerlach, C., Moseman, E.A., Loughhead, S.M., Alvarez, D., Zwijnenburg, A.J., Waanders, L., Garg, R., de la Torre, J.C., and von Andrian, U.H. (2016). The Chemokine Receptor CX3CR1 Defines Three Antigen-Experienced CD8 T Cell Subsets with Distinct Roles in Immune Surveillance and Homeostasis. Immunity *45*, 1270–1284.

65. Olson, J.A., McDonald-Hyman, C., Jameson, S.C., and Hamilton, S.E. (2013). Effector-like $CD8^+$ T cells in the memory population mediate potent protective immunity. Immunity *38*, 1250–1260.

66. Joshi, N.S., Cui, W., Chandele, A., Lee, H.K., Urso, D.R., Hagman, J., Gapin, L., Kaech, S.M., et al. (2007). Inflammation directs memory precursor and short-lived effector CD8(+) T cell fates via the graded expression of T-bet transcription factor. Immunity *27*, 281–295.

67. Voehringer, D., Blaser, C., Brawand, P., Raulet, D.H., Hanke, T., and Pircher, H. (2001). Viral infections induce abundant numbers of senescent CD8 T cells. J. Immunol. *167*, 4838–4843.

68. Hendriks, J., Gravestein, L.A., Tesselaar, K., van Lier, R.A., Schumacher, T.N., and Borst, J. (2000). CD27 is required for generation and long-term maintenance of T cell immunity. Nat. Immunol. *1*, 433–440.

69. Utzschneider, D.T., Charmoy, M., Chennupati, V., Pousse, L., Ferreira, D.P., Calderon-Copete, S., Danilo, M., Alfei, F., Hofmann, M., Wieland, D., et al. (2016). T Cell Factor 1-Expressing Memory-like CD8(+) T Cells Sustain the Immune Response to Chronic Viral Infections. Immunity *45*, 415–427.

70. Schuler, M.M., Nastke, M.D., and Stevanovikć, S. (2007). SYFPEITHI: database for searching and T-cell epitope prediction. Methods Mol. Biol. *409*, 75–93.

71. Falk, K., Rötzschke, O., Stevanović, S., Jung, G., and Rammensee, H.G. (1991). Allele-specific motifs revealed by sequencing of self-peptides eluted from MHC molecules. Nature *351*, 290–296.

72. Yu, W., Jiang, N., Ebert, P.J., Kidd, B.A., Müller, S., Lund, P.J., Juang, J., Adachi, K., Tse, T., Birnbaum, M.E., et al. (2015). Clonal Deletion Prunes but Does Not Eliminate Self-Specific $\alpha\beta$ CD8(+) T Lymphocytes. Immunity *42*, 929–941.

CellPress
OPEN ACCESS

73. McLane, L.M., Abdel-Hakeem, M.S., and Wherry, E.J. (2019). CD8 T Cell Exhaustion During Chronic Viral Infection and Cancer. Annu. Rev. Immunol. *37*, 457–495.

74. Speiser, D.E., Utzschneider, D.T., Oberle, S.G., Münz, C., Romero, P., and Zehn, D. (2014). T cell differentiation in chronic infection and cancer: functional adaptation or exhaustion? Nat. Rev. Immunol. *14*, 768–774.

75. Utzschneider, D.T., Legat, A., Fuertes Marraco, S.A., Carrié, L., Luescher, I., Speiser, D.E., and Zehn, D. (2013). T cells maintain an exhausted phenotype after antigen withdrawal and population reexpansion. Nat. Immunol. *14*, 603–610.

76. Larocca, C., and Schlom, J. (2011). Viral vector-based therapeutic cancer vaccines. Cancer J. *17*, 359–371.

77. Zhang, L., Bridle, B.W., Chen, L., Pol, J., Spaner, D., Boudreau, J.E., Rosen, A., Bassett, J.D., Lichty, B.D., Bramson, J.L., and Wan, Y. (2013). Delivery of viral-vectored vaccines by B cells represents a novel strategy to accelerate CD8(+) T-cell recall responses. Blood *121*, 2432–2439.

78. de Lamballerie, X., Fulhorst, C.F., and Charrel, R.N. (2007). Prevalence of antibodies to lymphocytic choriomeningitis virus in blood donors in southeastern France. Transfusion *47*, 172–173.

79. Lledó, L., Gegúndez, M.I., Saz, J.V., Bahamontes, N., and Beltrán, M. (2003). Lymphocytic choriomeningitis virus infection in a province of Spain: analysis of sera from the general population and wild rodents. J. Med. Virol. *70*, 273–275.

80. Elbers, A.R., Vecht, U., Osterhaus, A.D., Groen, J., Wisselink, H.J., Diepersloot, R.J., and Tielen, M.J. (1999). Low prevalence of antibodies against the zoonotic agents Brucella abortus, Leptospira spp., Streptococcus suis serotype II, hantavirus, and lymphocytic choriomeningitis virus among veterinarians and pig farmers in the southern part of The Netherlands. Vet. Q. *21*, 50–54.

81. Stephensen, C.B., Blount, S.R., Lanford, R.E., Holmes, K.V., Montali, R.J., Fleenor, M.E., and Shaw, J.F. (1992). Prevalence of serum antibodies against lymphocytic choriomeningitis virus in selected populations from two U.S. cities. J. Med. Virol. *38*, 27–31.

82. Borducchi, E.N., Cabral, C., Stephenson, K.E., Liu, J., Abbink, P., Ng'ang'a, D., Nkolola, J.P., Brinkman, A.L., Peter, L., Lee, B.C., et al. (2016). Ad26/MVA therapeutic vaccination with TLR7 stimulation in SIV-infected rhesus monkeys. Nature *540*, 284–287.

83. Roberts, D.M., Nanda, A., Havenga, M.J., Abbink, P., Lynch, D.M., Ewald, B.A., Liu, J., Thorner, A.R., Swanson, P.E., Gorgone, D.A., et al. (2006). Hexon-chimaeric adenovirus serotype 5 vectors circumvent pre-existing anti-vector immunity. Nature *441*, 239–243.

84. Smith, C.L., Mirza, F., Pasquetto, V., Tscharke, D.C., Palmowski, M.J., Dunbar, P.R., Sette, A., Harris, A.L., and Cerundolo, V. (2005). Immunodominance of poxviral-specific CTL in a human trial of recombinant-modified vaccinia Ankara. J. Immunol. *175*, 8431–8437.

85. Frahm, N., DeCamp, A.C., Friedrich, D.P., Carter, D.K., Defawe, O.D., Kublin, J.G., Casimiro, D.R., Duerr, A., Robertson, M.N., Buchbinder, S.P., et al. (2012). Human adenovirus-specific T cells modulate HIV-specific T cell responses to an Ad5-vectored HIV-1 vaccine. J. Clin. Invest. *122*, 359–367.

86. O'Brien, K.L., Liu, J., King, S.L., Sun, Y.H., Schmitz, J.E., Lifton, M.A., Hutnick, N.A., Betts, M.R., Dubey, S.A., Goudsmit, J., et al. (2009). Adenovirus-specific immunity after immunization with an Ad5 HIV-1 vaccine candidate in humans. Nat. Med. *15*, 873–875.

87. Leen, A.M., Christin, A., Khalil, M., Weiss, H., Gee, A.P., Brenner, M.K., Heslop, H.E., Rooney, C.M., and Bollard, C.M. (2008). Identification of hexon-specific CD4 and CD8 T-cell epitopes for vaccine and immunotherapy. J. Virol. *82*, 546–554.

88. Kedl, R.M., Rees, W.A., Hildeman, D.A., Schaefer, B., Mitchell, T., Kappler, J., and Marrack, P. (2000). T cells compete for access to antigen-bearing antigen-presenting cells. J. Exp. Med. *192*, 1105–1113.

89. Loyer, V., Fontaine, P., Pion, S., Hétu, F., Roy, D.C., and Perreault, C. (1999). The in vivo fate of APCs displaying minor H antigen and/or MHC differences is regulated by CTLs specific for immunodominant class I-associated epitopes. J. Immunol. *163*, 6462–6467.

90. Hollingsworth, R.E., and Jansen, K. (2019). Turning the corner on therapeutic cancer vaccines. NPJ Vaccines *4*, 7.

91. Battegay, M., Cooper, S., Althage, A., Bänziger, J., Hengartner, H., and Zinkernagel, R.M. (1991). Quantification of lymphocytic choriomeningitis virus with an immunological focus assay in 24- or 96-well plates. J. Virol. Methods *33*, 191–198.

92. Nakauchi, M., Fukushi, S., Saijo, M., Mizutani, T., Ure, A.E., Romanowski, V., Kurane, I., and Morikawa, S. (2009). Characterization of monoclonal antibodies to Junin virus nucleocapsid protein and application to the diagnosis of hemorrhagic fever caused by South American arenaviruses. Clin. Vaccine Immunol. *16*, 1132–1138.

93. Hufert, F.T., Lüdke, W., and Schmitz, H. (1989). Epitope mapping of the Lassa virus nucleoprotein using monoclonal anti-nucleocapsid antibodies. Arch. Virol. *106*, 201–212.

94. Probst, H.C., Tschannen, K., Gallimore, A., Martinic, M., Basler, M., Dumrese, T., Jones, E., and van den Broek, M.F. (2003). Immunodominance of an antiviral cytotoxic T cell response is shaped by the kinetics of viral protein expression. J. Immunol. *171*, 5415–5422.

95. Ludewig, B., Ochsenbein, A.F., Odermatt, B., Paulin, D., Hengartner, H., and Zinkernagel, R.M. (2000). Immunotherapy with dendritic cells directed against tumor antigens shared with normal host cells results in severe autoimmune disease. J. Exp. Med. *191*, 795–804.

96. Lin, K.Y., Guarnieri, F.G., Staveley-O'Carroll, K.F., Levitsky, H.I., August, J.T., Pardoll, D.M., and Wu, T.C. (1996). Treatment of established tumors with a novel vaccine that enhances major histocompatibility class II presentation of tumor antigen. Cancer Res. *56*, 21–26.

97. Bouckaert, R., Vaughan, T.G., Barido-Sottani, J., Duchêne, S., Fourment, M., Gavryushkina, A., Heled, J., Jones, G., Kühnert, D., De Maio, N., et al. (2019). BEAST 2.5: An advanced software platform for Bayesian evolutionary analysis. PLoS Comput. Biol. *15*, e1006650.

98. Grob, P., Schijns, V.E., van den Broek, M.F., Cox, S.P., Ackermann, M., and Suter, M. (1999). Role of the individual interferon systems and specific immunity in mice in controlling systemic dissemination of attenuated pseudorabies virus infection. J. Virol. *73*, 4748–4754.

99. Chen, J., Trounstine, M., Alt, F.W., Young, F., Kurahara, C., Loring, J.F., and Huszar, D. (1993). Immunoglobulin gene rearrangement in B cell deficient mice generated by targeted deletion of the JH locus. Int. Immunol. *5*, 647–656.

100. Townsend, M.J., Fallon, P.G., Matthews, D.J., Jolin, H.E., and McKenzie, A.N. (2000). T1/ST2-deficient mice demonstrate the importance of T1/ST2 in developing primary T helper cell type 2 responses. J. Exp. Med. *191*, 1069–1076.

101. Lan, S., McLay, L., Aronson, J., Ly, H., and Liang, Y. (2008). Genome comparison of virulent and avirulent strains of the Pichinde arenavirus. Arch. Virol. *153*, 1241–1250.

102. Pinschewer, D.D., Perez, M., Sanchez, A.B., and de la Torre, J.C. (2003). Recombinant lymphocytic choriomeningitis virus expressing vesicular stomatitis virus glycoprotein. Proc. Natl. Acad. Sci. USA *100*, 7895–7900.

103. FigTree (2018). Computer program distributed by author. http://tree.bio.ed.ac.uk/software/figtree/.

104. Duan, F., Simeone, S., Wu, R., Grady, J., Mandoiu, I., and Srivastava, P.K. (2012). Area under the curve as a tool to measure kinetics of tumor growth in experimental animals. J. Immunol. Methods *382*, 224–228.

STAR★METHODS

KEY RESOURCES TABLE

REAGENT or RESOURCE	SOURCE	IDENTIFIER
Antibodies		
Rat monoclonal anti-LCMV-NP: VL4	Dr. D.D. Pinschewer Battegay et al.[91]	PMID: 1939506
Rat monoclonal anti GFP	Biolegend	Cat# 338002; RRID: AB_ 1279414
mouse monoclonal anti PICV-NP: 17.2.E4-2	Nakauchi et al.[92]	PMID: 19553554
mouse monoclonal anti CAND-NP: 17.1.C6.0	Nakauchi et al.[92]	PMID: 19553554
mouse monoclonal anti MOPV-NP: 2B5	Hufert et al.[93]	PMID: 2476109
Rat monoclonal anti mouse CD8a; Clone 53-6.7	Biolegend	Cat# 562611
Rat monoclonal anti mouse CD45R/B220: Clone RA3-6B2	BD Biosciences	Cat# 553088
Syrian Hamster monoclonal anti mouse KLRG1; Clone 2F1/KLRG1	Biolegend	Cat# 138418
Rat monoclonal anti mouse CD127; clone A7R34	Biolegend	Cat# 135027
mouse monoclonal anti mouse CX3CR1; clone SA011F11	Biolegend	Cat# 149031
Armenian Hamster monoclonal anti mouse CD27; clone LG3A10	Biolegend	Cat# 124216
Rat monoclonal anti mouse CD43; clone 1B11	Biolegend	Cat# 121214
mouse monoclonal anti mouse Granzyme B; clone GB12	Thermo Fisher	Cat# MHGB04
Rat monoclonal anti mouse Ki-67; clone SolA15	Thermo Fisher	Cat# 56-5698-80
Rat monoclonal anti mouse IFNg; clone XMG1.2	Biolegend	Cat# 505810
Rat monoclonal anti mouse TNFa; clone MP6-XT22	Biolegend	Cat# 506324
Rat monoclonal anti mouse IL2; clone JES6-5H4	Biolegend	Cat# 503808
Rat monoclonal anti mouse CD11b; clone M1/70	BD Biosciences	Cat# 553310
Armenian Hamster monoclonal anti mouse CD11c; clone HL3	BD Biosciences	Cat# 553801
Rat monoclonal anti mouse CD19; clone 1D3	BD Biosciences	Cat# 553785
Rat monoclonal anti mouse NKp46; clone 29A1.4	Biolegend	Cat# 137605
Rat monoclonal anti mouse CD4; clone RM4-5	Biolegend	Cat# 100548
Rabbit monoclonal anti mouse TCF1; clone C63D9	Cell Signaling	Cat# 11/2014
Donkey polyclonal anti rabbit; Poly4046	Biolegend	Cat# 406410
Rat monoclonal anti mouse Eomes; clone Dan11mag	eBioscience	Cat# 50-4875-82
mouse monoclonal anti mouse T-bet; clone eBio4B10 (4B10)	Thermo Fisher	Cat# 12-5825-82; RRID: AB_925761

(Continued on next page)

CellPress
OPEN ACCESS

Continued

REAGENT or RESOURCE	SOURCE	IDENTIFIER
EnVision+ System-HRP Labeled Polymer	Dako	Cat# K4001
Bacterial and virus strains		
LCMV-WT	Sommerstein et al.[45]	PMID: 26587982
artLCMV-GFP/RFP	Kallert et al.[29]	PMID: 28548102
r3LCMV-GFP/RFP	Kallert et al.[29]	PMID: 28548102
artLCMV-P1A	Kallert et al.[29]	PMID: 28548102
artLCMV-E7E6	This paper	N/A
PICV-WT	This paper	N/A
artPICV-GFP/RFP	This paper	N/A
r3PICV-GFP/RFP	This paper	N/A
artPICV-P1A	This paper	N/A
artPICV-E7E6	This paper	N/A
CAND-WT	This paper	N/A
artCAND-GFP/RFP	This paper	N/A
r3CAND-GFP/RFP	This paper	N/A
artCAND-P1A	This paper	N/A
artCAND-E7E6	This paper	N/A
MOPV-WT	This paper	N/A
artMOPV-P1A	This paper	N/A
VACC-NP396	Probst et al.[94]	PMID: 14607945
VACC-lacZ	Ludewig et al.[95]	PMID: 10704461
Chemicals, peptides, and recombinant proteins		
P1A-Tetramer-PE: H2-Ld-LPYLGWLVF	Tetramer Core facility University Lausanne	TA P815 35-43 PE
LCMV-NP(396-404)-Tetramer-PE: H2-Db-FQPQNGQFI	NIH Tetramer Core Facility at Emory University	N/A
LCMV-NP(118-126)-Dextramers: H2-Ld-RPQASGVYM	Immudex, Denmark	JG2750-APC
HPV-E7(49-57)-Dextramers: H2-Db-RAHYNIVTF	Immudex, Denmark	JA2195-APC
VACC-B8R(20-27)Tetramer-PE: H-2Kb-TSYKFESV	Tetramer Core facility University Lausanne	MVA-B8R-20-27-PE
HPV-E7 peptide set: 22 peptides (peptide scan 15/11)	JPT	HPV16-E7 overlapping peptides
HPV-E6 peptide set: 37 peptides (peptide scan 15/11)	JPT	HPV16-E6 overlapping peptides
Lipofectamine 2000 Transfection Reagent	Thermo Fisher / Invitrogen	Cat# 11668019
TRI Reagent	Sigma Aldrich / Merck	T9424
Critical commercial assays		
QIAamp-Viral RNA-Mini	QIAGEN	Cat# 52906
QIAGEN OneStep RT-PCR Kit	QIAGEN	Cat# 21020
SuperScript IV First-Strand Synthesis System	Thermo Fisher	Cat# 18091050
Zombie UV Fixable Viability kit	Biolegend	Cat# 423108
eBioscienceFoxp3/ Transcription Factor Staining Buffer Set	Thermo Fisher	Cat# 00-5523-00
Experimental models: cell lines		
Hamster: BHK 21 (clone 13) cells	ECACC	Cat# 85011433 RRID: CVCL_1915
Hamster: BHK 21-GP cells	Flatz et al.[41]	PMID: 20139992
Human: HEK293T cells	ECACC	Cat# 12022001 RRID: CVCL_0063

(*Continued on next page*)

Continued		
REAGENT or RESOURCE	SOURCE	IDENTIFIER
Human: 293T-GP cells	Flatz et al.[41]	PMID: 20139992
Mouse: P815 cells	ATCC	Cat# TIB-64 RRID: CVCL_2154
Mouse: TC-1 cells	Lin et al.[96]	CVCL_4699 PMID: 8548765
African green monkey: BSC40 cells	ATCC	Cat#CRL-2761 RRID: CVCL_3656
Human: FreeStyle 293-F suspension cells	Invitrogen / ThermoFisher	Cat# R790-07
Mouse: NIH 3T3 cells	ATCC	Cat#CRL-1658 RRID::CVCL_0594
Experimental models: organisms/strains		
Mus musculus: C57BL/6J	Charles River	C57BL/6J (JAX Mice Strain)
Mus musculus: C57BL/6N	Charles River	C57BL/6NCrl
Mus musculus: AGRAG mice	Grob et al., 1999;	PMID: 10233935
Mus musculus: JHT mice	Chen et al., 1993;	PMID: 8347558
Mus musculus: Il1rl1$^{-/-}$ mice	Townsend et al., 2000;	PMID: 10727469
Mus musculus: BALB/c mice	Janvier Labs	Balb/cAnNRj
Mus musculus: DBA/2 mice	Janvier Labs	DBA/2Jrj
Cavia porcellus: Dunkin Hartley guinea pig	Charles River	Hartley Guinea Pig Crl:HA
Software and algorithms		
Prism 9.0.0	GraphPad	RRID: SCR_002798
QuickCalcs	GraphPad	RRID:SCR_000306
FlowJo 10.7.1	Becton Dickinson & Company	RRID: SCR_008520
Definiens Developer D Software	Definiens Inc. / MedImmune	N/A
Adobe Photoshop CS6	Adobe Photoshop	RRID: SCR_014199
BEAST 2 software	Bouckaert et al.[97]	PMID: 30958812
TreeAnnotator software: FigTree v1.4.4	FigTree	N/A
Deposited data		
Raw and analyzed data	This paper	https://doi.org/10.5281/zenodo.4488070

RESOURCE AVAILABILITY

Lead contact

Further information and requests for resources and reagents should be directed to the Lead Contact, Daniel D. Pinschewer (Daniel.Pinschewer@unibas.ch).

Materials availability

Material transfer agreements with standard academic terms will be established to document reagent sharing by the lead contact's institution. artPICV vector materials will be supplied by Hookipa Biotech GmbH under MTA."

Data and code availability

The accession number for the data reported in this study is Zenodo: https://doi.org/10.5281/zenodo.4488070.

Oligonucleotide primer sequences are available from the authors upon request.

EXPERIMENTAL MODEL AND SUBJECT DETAILS

Animals and ethics statement

AGRAG mice (*IFNα/βR*$^{-/-}$, *IFNγR*$^{-/-}$, *RAG1*$^{-/-}$ triple-deficient),[98] B cell-deficient JHT mice[99] and ST2-deficient *Il1rl1*$^{-/-}$ mice on C57BL/6J background have been described[31,100] and they were bred at the Laboratory Animal Sciences Center (LASC) of the University of Zurich, Switzerland. C57BL/6J, BALB/c and DBA/2 wild-type mice were either purchased from Charles River and Janvier Labs or were bred at LASC and at the University of Geneva, Switzerland, under specific pathogen-free (SPF) conditions.

TC-1 tumor therapy studies in mice were performed at Hookipa Biotech GmbH using C57BL/6N mice purchased from Charles River, Sulzfeld, Germany. These experiments were approved by the Austrian authorities and were carried out in accordance with the approved guidelines for animal experiments at Hookipa Biotech GmbH.

All other mouse experiments were performed at the Universities of Basel and Geneva in accordance with the Swiss law for animal protection. Permission was granted by the Veterinäramt Basel-Stadt and by the Direction générale de la santé, Domaine de l'expérimentation animale, of the Canton of Geneva, respectively. Animals in experimental groups were sex- and age-matched. In general, adult animals of both genders were used to reduce the number of animals bred for research purposes. P1A-specific immunogenicity assessments and P815 tumor control studies were conducted in female mice. Mice in tumor therapy experiments were assigned to groups in a manner to assure even distribution of tumor volumes between groups at the time of tumor therapy. In accordance with the Swiss law for animal protection mice exhibiting wounds on the tumor or displaying signs of distress (evident namely in lethargy, hunchback, piloerection, emaciation and agonal breathing) were immediately euthanized irrespective of tumor size and diameter. Study sample sizes in animal experiments were chosen based on experience in our labs with respect to group sizes readily revealing biologically significant differences in the experimental models used. The groups were neither randomized nor were experiments conducted in a blinded fashion.

The PICV virulence study in guinea pigs was conducted at Meditox (Czech Republic) and was approved by the Institutional Animal Care and Use Committee (IACUC) and the Committee for Animal Protection of the Ministry of Health of the Czech Republic. Dunkin Hartley guinea pigs were from Charles River, France, and weighed 370 – 520 g at the start of the study.

Cell lines

BHK-21 cells, HEK293T cells were purchased from ECACC (Clone 13, Cat #85011433), P815 mastocytoma cells (TIB-64), NIH 3T3 and BSC40 cells from ATCC. FreeStyle 293-F suspension culture cells were purchased from Invitrogen/ThermoFisher. LCMV-GP-expressing BHK-21 cells (BHK-21-GP) and 293T-GP cells have previously been described.[41] All cell lines were regularly tested for mycoplasma and were negative. Owing to their origin from renowned international repositories and vendors they were not authenticated.

METHOD DETAILS

Viruses, titration and neutralization test

The titration of LCMV, PICV, MOPV and derived vectors by immunofocus assay has been described[29,91] and was performed using NIH 3T3 cells as a substrate, CAND and derived vectors were titrated using HEK293T cells by analogous techniques. For detection of GFP-expressing artPICV and r3PICV infectivity by immunofocus assay we used rat-anti-GFP antibody (Biolegend).[29] To quantify PICV and derived vectors by immunofocus assays, monoclonal antibody (mAb) 17.2.E4-2 [95] served as primary antibody. mAb 17.1.C6-9[92] was used for detection of CAND and mAb 2B5[93] for MOPV. LCMV, PICV and derived vectors batches were produced on BHK-21 cells and 293F cells, MOPV and artMOPV-P1A on BHK-21, CAND and derived vectors on 293T cells.

Recombinant vaccinia viruses expressing the NP396 miniepitope or lacZ, respectively (VACC-NP396, VACC-lacZ) have been described.[94,95] They were grown and titrated on BSC40 cells.

The neutralizing capacity of immune serum was determined by immunofocus reduction assays.[91]

Viral virulence testing

Intracranial LCMV infection was administered through the skull and mice developing signs of terminal disease were euthanized in accordance with the Swiss law.

The wellbeing of guinea pigs undergoing PICV or artPICV infection was monitored twice daily during the entire study and clinically scored. Moribund animals were sacrificed. Humane endpoints were hypothermia (body temperature $< 35^\circ$C, determined at two independent monitoring time points) and/or body weight loss $\geqq 20\%$.

Tumor implantation and tumor measurement

P815 cells (10^6 per mouse) were implanted subcutaneously in the right flank. Tumor growth was assessed three times per week. The longest and the shortest diameter were determined using a caliper. Tumor volumes (mm^3) were calculated as ½ (length*width^2). When tumor volumes exceeded 1500 mm^3 or when the longest median tumor diameter exceeded 20 mm, mice were euthanized in accordance with the Swiss law.

TC-1 cells expressing HPV 16 E6 and E7[96] were obtained from Johns Hopkins University. For tumor implantation, 10^5 cells were injected subcutaneously into the flank of C57BL/6 mice and, in accordance with the Austrian law, the experiment was terminated when tumor sizes exceeded 20 mm in any dimension.

Virus engineering, infection and immunization

The reverse genetic engineering of LCMV-WT, r3LCMV and artLCMV vectors using a polymerase I- / polymerase II-based plasmid system has been described.[39] PICV-based, CAND-based and MOPV-based vectors and the corresponding cDNA-derived WT viruses were generated using analogous expression cassettes and transfection procedures using BHK-21-GP cells as a cell substrate. In brief, we transfected 5×10^5 cells, seeded the day before into an M6 cell culture well, with 0.8 µg of each pol-I-driven S segment expression plasmid, 1 µg of pol-I-driven L segment, 1.4 µg of pol-II-driven L ORF expression plasmid and 0.8 µg of pol-II-driven NP expression plasmid using 12 µl Lipofectamine 2000. Six hours after transfection for the rescue of CAND and derived vectors,

10^5 293T-GP cells were added to each well. 72 hours after transfection, the cells were trypsinized and transferred to a T75 tissue culture flask. Virus- and vector-containing supernatants, respectively, were harvested 6-10 days after transfection. Wild-type CAND virus serving as a template for vector generation was generously provided by R. Charrel, Marseille, France. Its sequence was determined by RT-PCR Sanger sequencing and was identical to GenBank accession numbers HQ126698 and HQ126699. GenBank accession numbers EF529747.1 and EF529746.1 of the guinea pig-virulent PICV strain p18[101] were used for vector generation. Silent point mutations were designed into ORFs to delete BsmBI, BbsI and BamHI restriction sites, enabling molecular cloning strategies for transgene insertion as described.[102] cDNAs encoding for the L and NP ORFs as well as for the full-length L and S segments of CAND and PICV were synthesized by Genscript, the Netherlands, and were ligated into polymerase-II- and polymerase-I-driven expression cassettes, respectively.[39] MOPV cDNAs for virus and vector rescue (GenBank accession numbers JN561685.1 and JN561684.1) were generated by RT-PCR cloning (L and WT S segment, NP and L ORFs) and by gene synthesis (transgenic S segments of vectors). cDNAs of the full-length cancer-testis antigen P1A (comprising the immunodominant LPYLGWLVF epitope), a non-oncogenic fusion protein consisting of the complete HPV16 E7 and E6 sequences (comprising the immunodominant epitope RAHYNIVTF),[55] GFP or Tomato (TOM), were used for insertion into the respective vectors and viruses using a seamless cloning strategy previously described in detail.[102]

Infections and immunizations of mice with arenaviruses and arenavirus-based vectors were performed at a dose of 10e5 PFU i.v. unless specified otherwise. Vaccinia virus vectors were given at an intravenous dose of 2x10e6 PFU.

Assessment of blood-brain-barrier integrity

Blood-brain-barrier leakage was assessed by detecting IgG deposits in mouse brain parenchyma.

Cryosections of 10μm were fixed with 4% PFA for 15 min, endogenous peroxidases were inactivated and tissue sections were incubated with HRP-labeled-anti-mouse-IgG (Dako, K4001). Bound peroxidase polymers were visualized using polymerized 3.3′-diaminobenzidine (DAB, Dako, K5001). The stained sections were scanned using a Panoramic Digital Slide Scanner 250 Flash II at 200x magnification. Quantifications were performed on scanned slides applying a custom-programmed script to detect the DAB^+ area in Cognition Network Language (Definiens Developer D software). For representative images, white balance was adjusted and contrast was linearly enhanced using the tools "levels," "curves," "brightness" and "contrast" in Photoshop CS6 (Adobe).

Virus sequencing and genealogy tree building

Viral RNA was extracted from cell culture supernatant and from serum of infected mice using the QIAamp Viral RNA Mini Kit (QIAGEN, Cat No. 52906). RNA from spleens of mice was extracted using Tri Reagent (Sigma Aldrich). Reverse-transcription PCR was performed with the One Step RT-PCR kit (QIAGEN) and gene-specific primers. Amplified products were gel-purified for Sanger sequencing (Microsynth).

A mammarenavirus genealogy tree was built based on S segment sequences of the following viruses and GenBank accession numbers: Allpahuayo virus (AY081210.1), Amapari virus (AF485256.1), Junin virus (AY358023), Bear Canyon virus (AY924391), Sabia virus (U41071), Pichinde virus (K02734), Chapare virus (EU260463), Cupixi virus (AF512832), Flexal virus (AF512831), Gairo virus (KJ855308), Guanarito virus (AY129247), Ippy virus (DQ328877), Lassa virus (AF181854.1), Latino virus (AF512830), Loei River virus (KC669698), Lujo virus (FJ952384), Luna virus (AB586644), Lunk virus (AB693150), Machupo virus (AY129248), Mariental virus (KM272987), Merino Walk virus (GU078660), Mobala virus (AY342390), Mopeia virus (AY772170), Okahandja virus (KM272988), Oliveros virus (U34248), Parana virus (AF485261), Pirital virus (AF485262), Ryukyu virus (KM020191), Solwezi virus (AB972428), Souris virus (KP050227), Tacaribe virus (M20304), Tamiami virus (AF485263), Wenzhou virus (KJ909794), Whitewater Arroyo virus (AF228063). To build the phylogenetic tree, we used the software package BEAST2[97] with a TN93 site model (the corresponding .xml -file will be deposited for further reference). The MCMC chain ran for 10'000'000 steps. All ESS were well above the critical threshold. The maximum credibility tree was constructed with TreeAnnotator and the phylogeny displayed with FigTree v1.4.4.[103]

Flow cytometry

Antibodies against CD8 (53-6.7 or Ly-3.2), CD45R/B220 (RA3-6B2), Klrg1 (2F1), CD127 (A7R34), CX3CR1 (SA011F11), CD27 (LG3A10), CD43 (1B11), GrzB (GB12), Ki67 (solA15), IFN-γ (XMG1.2), TNF (MP6-XT22), IL-2 (JES6-5H4) CD11b (M1/70) CD11c (HL3) CD19(1D3) Nkp46 (29A1.4) and CD4 (RM4-5) were from Biolegend, BD Biosciences/PharMingen and eBioscience/ThermoFisher. To assess intracellular levels of the transcription factor Tcf1, primary antibody binding (C63D8, Cell Signaling) was detected using donkey anti-rabbit IgG PE (Poly4064-eBisocience). Eomes (Dan11mag) and T-bet (4B10) were detected using the eBioscience™ FOXP3 transcription factor staining kit (Invitrogen).

Dead cells were excluded with Zombie UV Fixable Viability Kit (Biolegend, Cat. #423108). P1A epitope- (LPYLGWLVF), B8R epitope- (TSYKFESV) and NP396 epitope- (FQPQNGQFI) specific CTLs were identified by peptide-MHC class I tetramers after gating on $CD8^+B220^-$ lymphocytes. The H-$2D^b$ tetramer loaded with the NP396 epitope and conjugated to PE was obtained through the NIH Tetramer Core Facility, the H-$2L^d$ tetramer loaded with the P1A epitope and conjugated to PE as well as the H-$2K^b$ tetramer loaded with the B8R epitope and conjugated to PE were purchased from the University of Lausanne Tetramer core facility. For tumor-infiltrating CTL analyses, cells expressing CD11b, CD11c, CD19 or NKp46 were excluded. For detection of E7- (RAHYNIVTF epitope) and NP118- (RPQASGVYM epitope) specific CTLs the corresponding dextramers (Immundex) were used analogously (for simplicity referred to as "tetramer" in the text). Splenic single-cell suspensions were prepared by mechanical disruption and were counted

using the respective single-use chambers in a Immunospot S6 device (C.T.L.). Total numbers of peptide-MHC tetramer-binding CTLs were back calculated. Cytokine profiles after restimulation with overlapping peptide sets spanning the E7 and E6 proteins of HPV16, respectively (JPT) were determined in intracellular cytokine assays as previously described.[31] Samples were measured on BD LSRFortessa flow cytometers and were analyzed using FlowJo software (Becton Dickinson).

For TIL analysis, tumors were dissected and digested with accutase (Sigma Aldrich), Collagenase IV (Worthington), Hyaluronidase (Sigma Aldrich) and DNaseI (Sigma Aldrich) for 60 min at 37°C, followed by red blood cell lysis. Single cell suspensions were filtered using a cell strainer (70 μM).

QUANTIFICATION AND STATISTICAL ANALYSIS

Statistical testing

For statistical analysis, GraphPad Prism software (Version 9.0, GraphPad Software) was used unless stated otherwise. Differences between two groups were generally assessed using unpaired two-tailed Student's t tests. To compare one group against multiple other groups we used one-way Analysis Of Variance (ANOVA), followed by Dunnett's post hoc test (Figures 7G–7J). Two-way ANOVA with Sidak's post-test was used to compare multiple cytokine-producing T cell subpopulations of two groups.

Tumor volume curves were compared as described.[104] In brief, the area under the curve (AUC) for each individual animal was calculated using GraphPad Prism software and groups were compared pairwise by two-tailed Mann-Whitney tests. To calculate AUCs for all groups and animals throughout the 41d and 60d periods in Figures 7A and 7B, respectively, animals reaching humane endpoints of tumor volume were assigned either the maximally permitted tumor volume (1500 mm^3, termination criterion for Figure 7A) or the maximally reached tumor (2815 mm^3 for Figure 7B) for time points after sacrifice. For comparison of long-term survival rates chi-square tests were performed using the GraphPad QuickCalcs online tool.

*P value*s of $p < 0.05$ were considered significant (*), $p < 0.01$ (**) as highly significant.

Cell Reports Medicine, Volume *2*

Supplemental information

Heterologous arenavirus vector prime-boost overrules self-tolerance for efficient tumor-specific CD8 T cell attack

Weldy V. Bonilla, Nicole Kirchhammer, Anna-Friederike Marx, Sandra M. Kallert, Magdalena A. Krzyzaniak, Min Lu, Stéphanie Darbre, Sarah Schmidt, Josipa Raguz, Ursula Berka, Ilena Vincenti, Mindaugas Pauzuolis, Romy Kerber, Sabine Hoepner, Stephan Günther, Carsten Magnus, Doron Merkler, Klaus K. Orlinger, Alfred Zippelius, and Daniel D. Pinschewer

Supplementary Figure 1

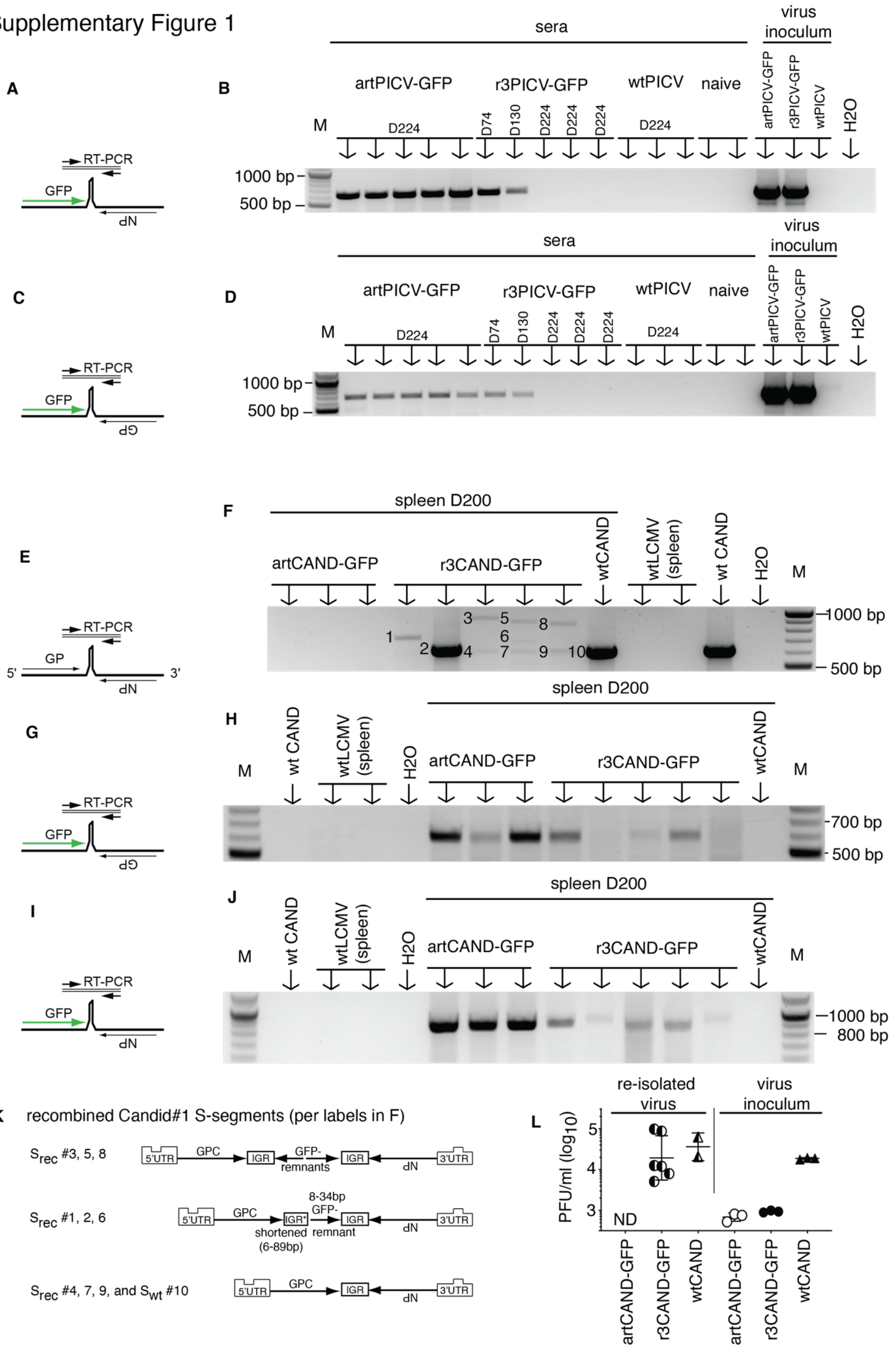

Figure S1: Genetic and phenotypic stability of artARENA and r3ARENA vectors. Related to Figure 1.

A-D: RT-PCR strategy (A) to amplify non-recombined S_{NP} (B) and RT-PCR strategy (C) to amplify S_{GP} segment (D) RNA species of both artPICV-GFP and r3PICV-GFP from the samples of the experiment shown in Fig. 1H-N. Gel electrophoresis image of RT-PCR products (B,D) obtained from serum samples collected on the indicated time points. Each lane represents an individual mouse. Sera from uninfected mice ("naive"), from three wtPICV infected mice and water were included as negative controls. Virus inocula served as reference.

E-L: We infected AGRAG mice with artCAND-GFP, r3CAND-GFP or CANDwt and collected spleen tissue 200 days later.

E,F: RT-PCR strategy (E) applied to amplify recombined wildtype-like CAND S segment RNA species re-uniting NP and GP sequences (F). Gel electrophoresis image of RT-PCR products (F) obtained from spleen samples collected on the indicated time points after infection of AGRAG mice. Water and spleen tissue of AGRAG mice infected with LCMVwt served as negative control. Spleen tissue from a wtCAND-inoculated AGRAG mouse and wtCAND inoculum virus served as positive controls. Each lane with a spleen sample represents an individual mouse.

G-J: RT-PCR strategy (G) to amplify non-recombined S_{GP} (H) and RT-PCR strategy (I) to amplify S_{NP} segment (J) RNA species of both artCAND-GFP and r3CAND-GFP. Gel electrophoresis image of RT-PCR products (H,J) obtained from spleen samples collected on d200. Each lane represents an individual mouse. Spleen tissue from AGRAG mice infected with wtCAND or wtLCMV as well as wtCAND virus from cell culture and water were included as negative controls in the assay.

K: Sequence analysis of the bands numbered in (F) suggested viral recombination products as schematically depicted in (K). IGR: S segment intergenic region; IGR*: truncated S segment intergenic region; UTR: untranslated region

L: 293T cells were infected at MOI=0.001 with viruses re-isolated from individual AGRAG mice on d200 of the experiment in (E-K) or with the inoculum used to infect the animals. Titers after 72h are shown. Symbols represent individual viral cultures from one mouse each (re-isolated virus) or from replicate cultures (virus inoculum). Mean±SD is indicated. We failed to re-isolate infectious virus from artCAND-infected mice (ND).

Supplementary Figure 2

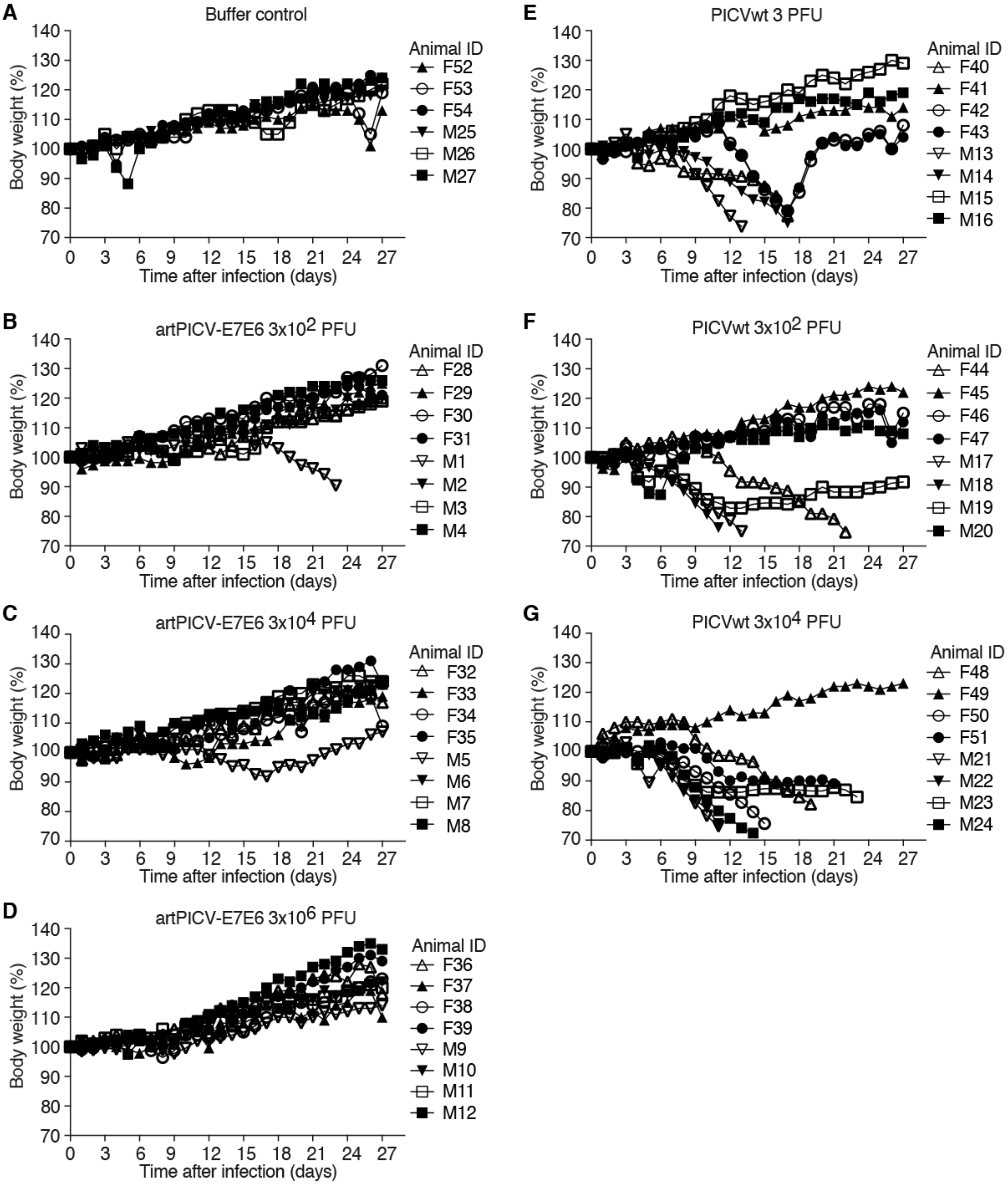

Figure S2: artARENA vectors are attenuated in guinea pigs. Related to Figure 2.

We infected groups of 8 adult Hartley guinea pigs, four of each sex (F: female; M: male), with either artPICV-E7E6 or PICVwt at the indicated doses intraperitoneally and monitored body weight over time. A group of six control animals (three of each sex) was administered diluent. Data are from the same experiment as in Fig. 2A-H.

Supplementary Figure 3

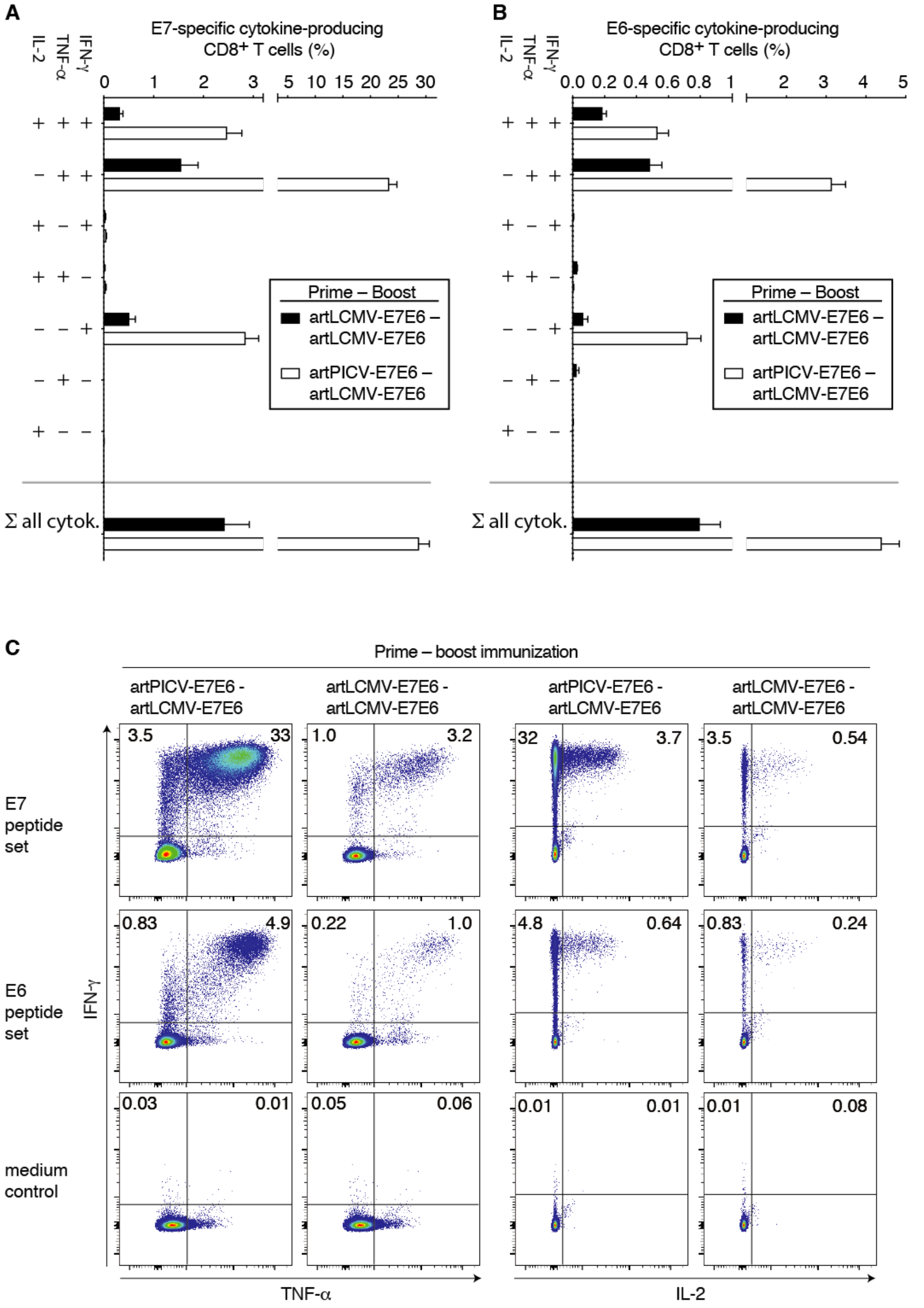

Figure S3: Functionality of CD8 T cell response to heterologous artARENA prime – boost vaccination. Related to Figure 3.

A-E: On d0 and d13 C57BL/6 mice were given i.v. vaccination with artPICV-E7E6 and artLCMV-E7E6, either as homologous or heterologous prime – boost, as indicated. On d51 the animals were sacrificed to determine E7- (A) and E6-specific (B) cytokine-secreting cells in spleen, respectively. Cytokine secretion was measured by intracellular cytokine staining upon restimulation with overlapping peptide sets spanning the E7 and E6 proteins, respectively. Bars represent the mean±SEM of 5 mice per group. Representative FACS plots are shown in (C). Numbers indicate the percentage of cells falling into the respective quadrants.

Supplementary Figure 4

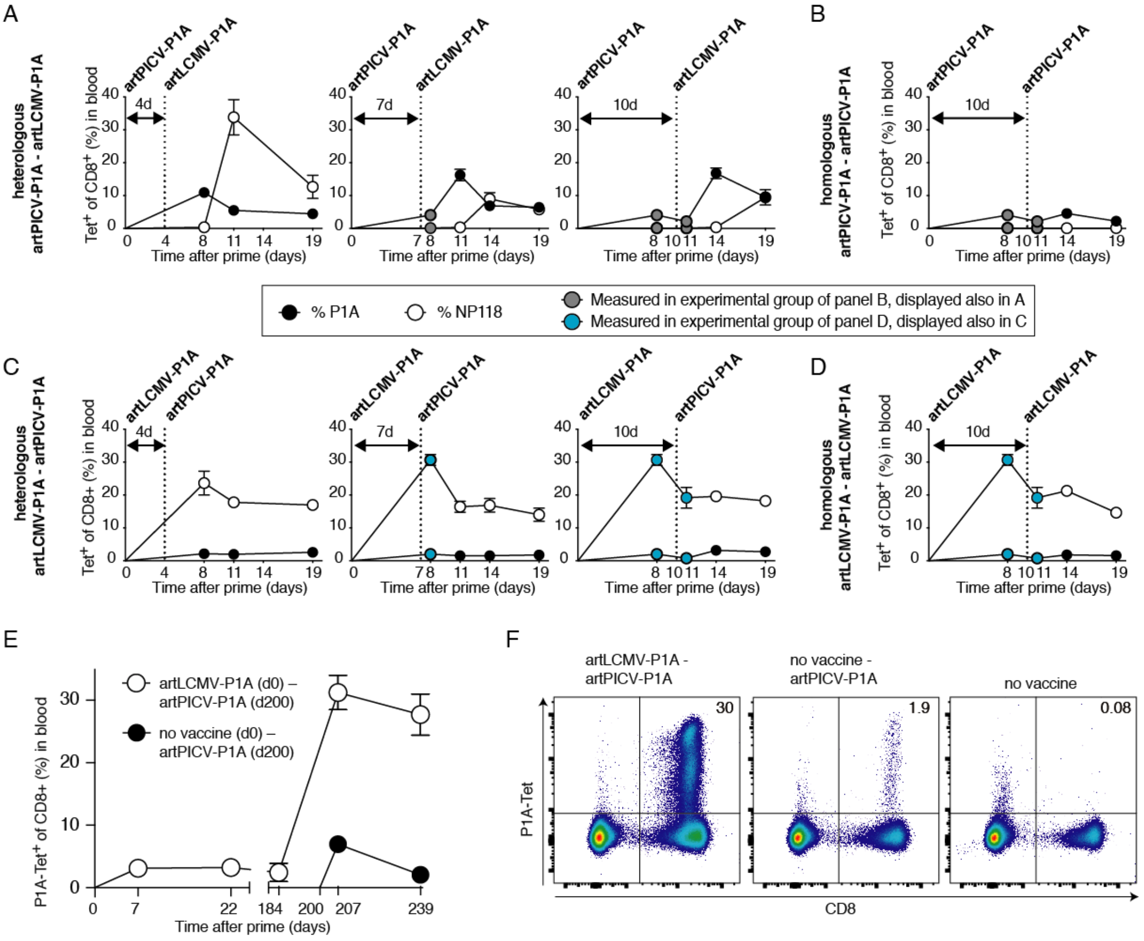

Figure S4: Impact of heterologous artARENA vector prime – boost interval on CTL responses, and efficient artPICV boost of artLCMV-primed responses after a long interval. Related to Figure 3.

A-D: We immunized DBA/2 mice using artLCMV-P1A and artPICV-P1A in heterologous (A,C) or homologous (B,D) prime – boost combinations and at intervals of 4, 7 or 10 days as indicated. P1A-specific and NP118-specific CTL responses in peripheral blood were determined at the indicated time points. Grey and blue symbols are included in the graphs to panels (A,C) for reference only: Grey symbols in (A,B) indicate values determined in the group displayed in (B) or upon single artPICV-P1A prime, blue symbols in (C,D) indicate values determined in the group displayed in (D) or upon single artLCMV-P1A prime. Symbols represent the mean±SEM of n=3-4 mice per group.

E-F: We immunized BALB/c mice with artLCMV-P1A on d0 and left controls without immunization. On d200 both groups were given artPICV-P1A for booster or prime immunization, respectively. P1A-specific CD8$^+$ T cell frequencies in peripheral blood were determined over time. Symbols in (E) represent the mean±SD of n=3 mice per group. Representative FACS plots from d239 are shown in (F), including also a naïve mouse as technical control (“no vaccine”). Values in (F) indicate the percentage of P1A-tetramer-binding cells amongst CD8$^+$ T cells.

Supplementary Figure 5

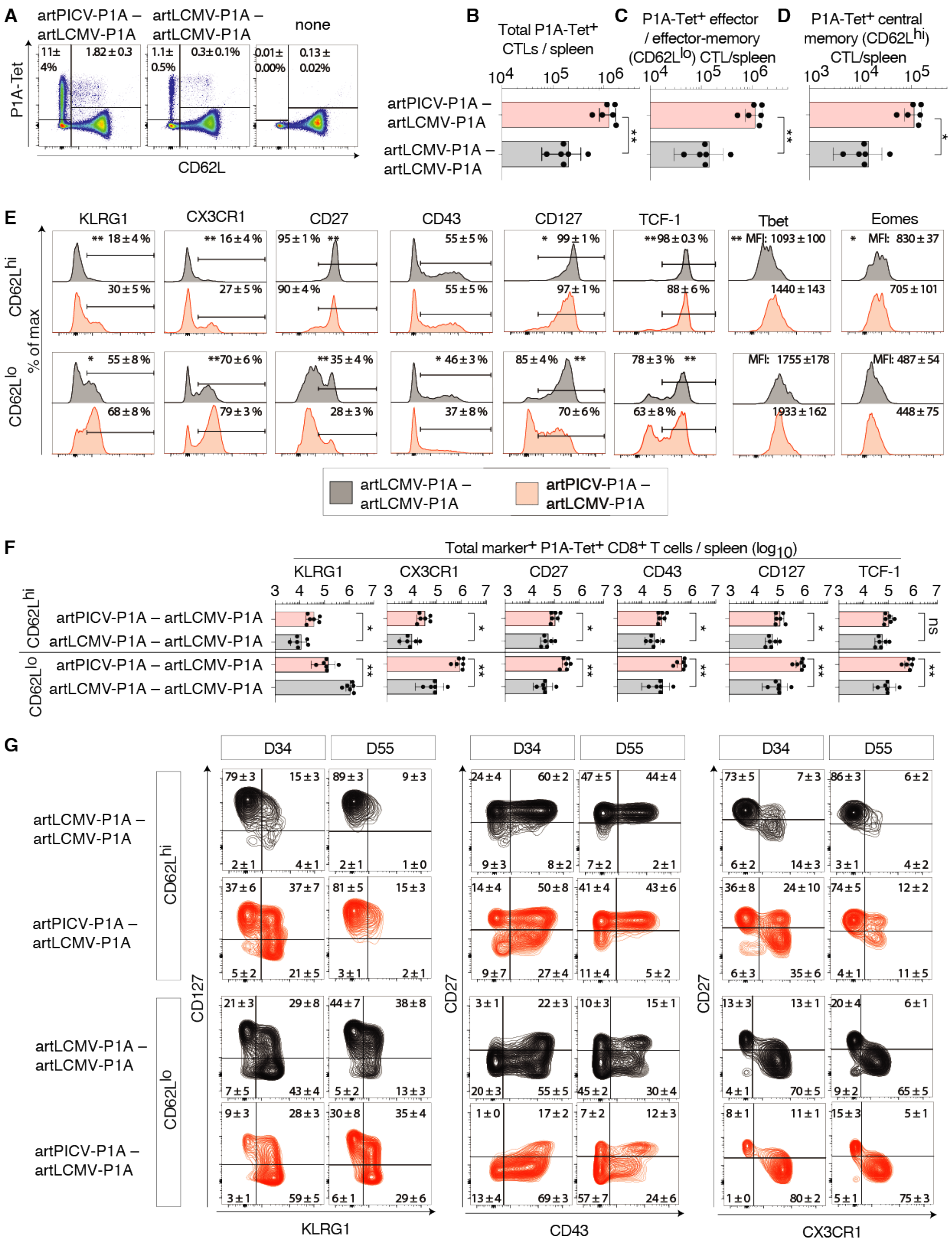

Figure S5: Phenotype of artARENA-induced CTLs one month after boost. Related to Figure 4.

We immunized BALB/c mice with artPICV-P1A and artLCMV-P1A in homologous or heterologous prime – boost vaccination i.v. on d0 and d27. On d34 (G, same experimental data set as shown in Fig. 4A-F) and d55 (A-G) we analyzed P1A-Tet-binding and CD62L expression by splenic CD8^{+} T cells (A, gated on CD8^{+}B220^{-}

lymphocytes). Unimmunized control mice are shown for comparison in (A) only. Numbers in (A) indicate the percentage of cells in the respective quadrant. Total P1A-Tet^{+} CTLs (B), P1A-specific effector/effector-memory CTLs (CD62L^{lo}, C) and P1A-specific central memory CTLs (CD62L^{hi}, D) were enumerated in spleen. In both subsets of P1A-specific CTLs, CD62L^{hi} and CD62L^{lo}, we determined the surface expression of KLRG1, CX3CR1, CD27, CD43 and CD127 as well as the master transcription factors Tcf-1, Tbet and Eomes (E). Total numbers of marker-expressing P1A-specific CTLs were enumerated in (F). Co-expression of CD127, KLRG1, CD27, CD43 and CX3CR1 in combinations as indicated are shown in (G). (A) shows representative FACS plots from individual mice, (G) shows combined events from 6 mice. Symbols in (B-D,F) represent individual mice, bars in (B-D,F) indicate the mean±SD. Numbers in (A,E,G) indicate the percentage of gated cells (mean±SD) or the mean fluorescence intensity (MFI±SD). Means were calculated from six mice per immunization group (A-G) or from three unimmunized controls (A). ** $p<0.01$, * $p<0.05$ by unpaired two-tailed Student's *t* test.

Supplementary figure 6

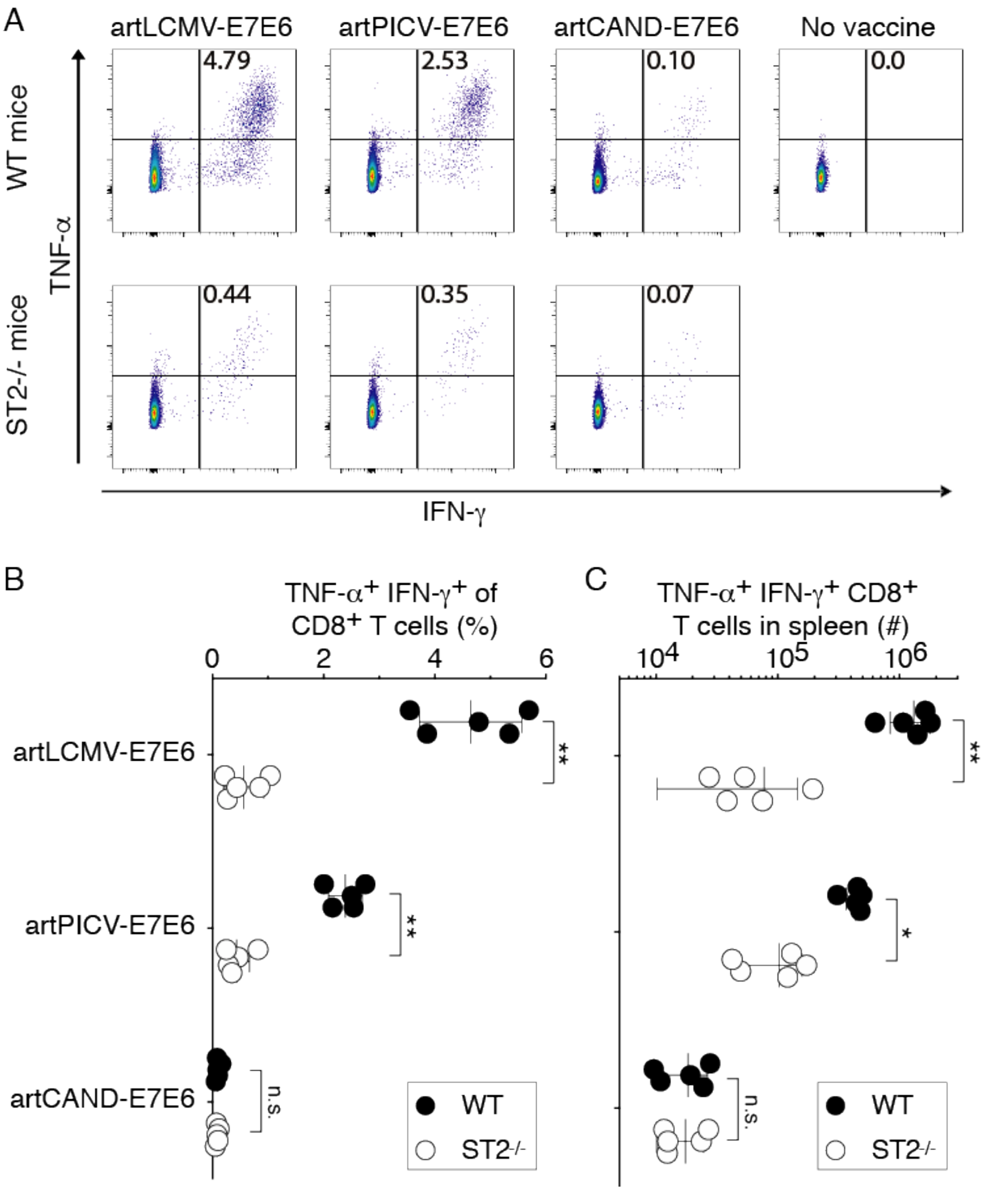

Figure S6: Dependence of artARENA-induced CTL responses on IL-33 – ST2 alarmin signaling. Related to Figure 4.

We immunized ST2-/- and wt mice on d0 with artLCMV-E7E6, artPICV-E7E6 or arCAND-E7E6 i.v. Wt controls were left unimmunized ("no vaccine"). $E7_{49\text{-}57}$ peptide-specific cytokine-secreting cells were determined on d9.

A: Representative FACS plots are shown.

B,C: TNF-α and IFN-γ co-producing $CD8^+$ T cells were enumerated and expressed as percentage of the total splenic $CD8^+$ T cell compartment (B) or as absolute number per spleen (C). Symbols in (B,C) represent individual mice with mean±SD. N=2. ** $p<0.01$ by two-way ANOVA with Sidak's post-test.

Supplementary figure 7

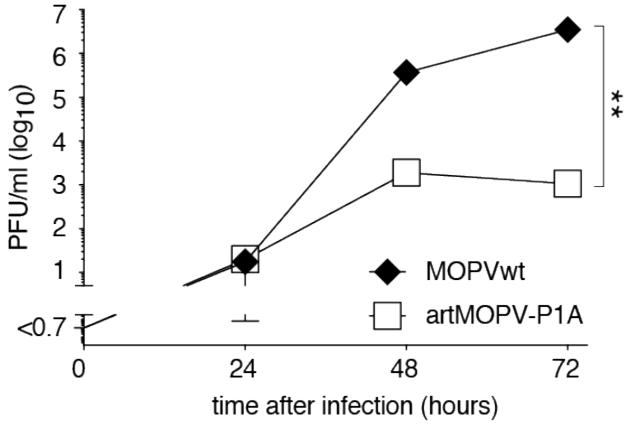

Figure S7: **Cell culture growth curve of artMOPV-P1A. Related to Figure 5.**

We infected BHK-21 cells at MOI=0.01 with artMOPV-P1A or with MOPVwt and determined infectious titers in the supernatant at the indicated time points. Symbols represent the mean±SD of three independent cell culture wells (error bars mostly hidden within the symbol size). ** p<0.01 by unpaired two-tailed Student's *t* test.
